# Time-of-day variation in motivation for everyday behaviors: A cluster-based approach to understanding individual differences

**DOI:** 10.1016/j.isci.2026.117366

**Published:** 2026-09-07

**Authors:** John Axelsson, Roshan Cools, Leonie J.T. Balter

**Affiliations:** 1 Department of Psychiatry, Radboud University Nijmegen Medical Centre, Nijmegen, the Netherlands; 2 Donders Institute for Brain, Cognition and Behaviour, Radboud University Nijmegen, Nijmegen, the Netherlands; 3 Department of Clinical Neuroscience, Karolinska Institutet, 171 65 Stockholm, Sweden; 4 Department of Psychology, Stress Research Institute, Stockholm University, 114 19 Stockholm, Sweden

**Keywords:** motivation, phenotypes, diurnal patterns, ecological momentary assessment, health behavior

## Abstract

Motivation governs everyday behavior and is often disrupted in psychiatric conditions, yet how motivation varies within the day, and how these patterns differ between individuals remains unclear. Here, we examined diurnal patterns of momentary subjective motivation for everyday activities, their translation into behavior, and how these patterns vary by trait-based measures linked to motivation. At baseline, 538 participants (424 women, 112 men, 2 non-binary, aged 18–77 years) completed questionnaires indexing psychiatric traits associated with altered motivation, revealing four clusters differing in appetitive motivation, and depressive features with varying levels of goal-directed behavior. Participants (*n* = 410) then completed ecological momentary assessments between ∼08:00 and 23:00, reporting momentary motivation for, and recent engagement in, everyday behaviors. Across clusters, motivation for recovery (rest, being alone) outweighed motivation for activity (socializing and exercise), creating a “motivational gap” (defined as the relative difference between recovery-oriented and activity-oriented motivations) that was largest in the morning and late evening and smallest around mid-day, with cluster-specific differences. Momentary motivation translated into subsequent behavior, with the strength of this coupling varying by time-of-day and motivational cluster. These findings identify diurnal motivation as an underrecognized source of individual variabilit in motivational state and behavioral.

## Highlights

• Four motivational phenotypes captured heterogeneity in everyday motivation
• Rest-oriented motivations outweighed activity motivations across the day
• The motivational gap varied systematically across the day and between phenotypes
• Motivation predicted behavior, with coupling varying by time of day and phenotype

## Graphical abstract

**Figure:**
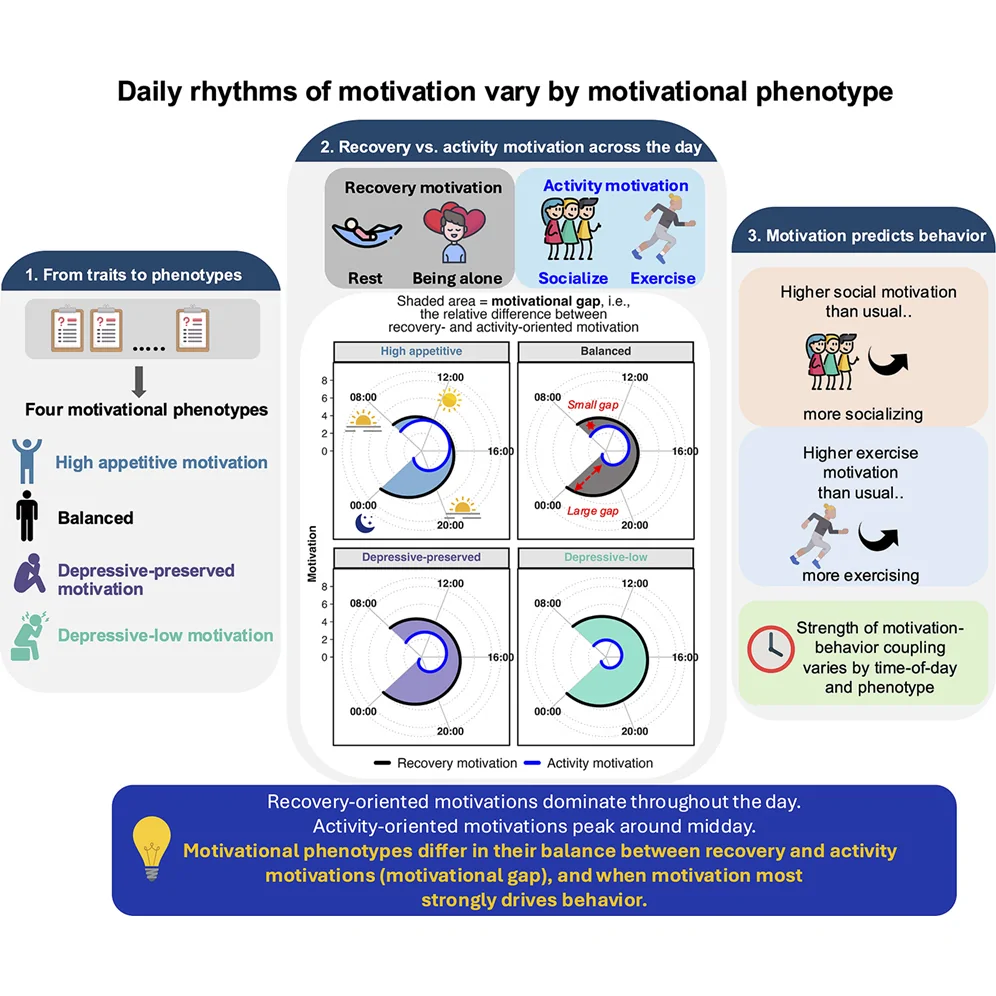

## Teaser

Behavioral neuroscience; Clinical neuroscience; Cognitive neuroscience

## Introduction

Motivation integrates the selection of goals and the mobilization of effort required to pursue them.^1^ Motivational dysregulation, whether manifesting as diminished goal-directed behavior, excessive motivation, or misdirected motivation toward non-optimal goals, is a hallmark of various psychiatric conditions. Conceptually, motivation can be viewed as a continuum ranging from hypo-to hyper-motivation. At the hypomotivation end, apathy, a syndrome of reduced goal-directed behavior, is common in depression and schizophrenia and is linked to poor functional outcomes.^2^^,^^3^^,^^4^ At the opposite end, hyper-motivation may manifest as steep delay discounting (valuing immediate over delayed rewards), or as misdirected motivation, presenting clinically as mania, attention-deficit hyperactivity disorder (ADHD), or substance-related problems.^5^^,^^6^^,^^7^ Notably, meaningful variation also exists below psychiatric diagnostic thresholds. Subclinical individual differences in reward sensitivity and approach-avoidance tendencies relate to anxiety, mood, attention, and substance use problems.^8^^,^^9^

Contemporary accounts frame motivation as a cost-benefit arbitration in which the brain evaluates the expected value of an action against effort costs and the opportunity cost of alternative actions.^10^^,^^11^ This arbitration shapes the vigor and the persistence of an action, and is likely time- and context-dependent. Across the day, variations in arousal, fatigue, and situational opportunity may dynamically shift the balance between value and effort. For example, physical activity may be more attractive when energy levels are higher, whereas rest becomes increasingly valuable toward the evening when homeostatic sleep pressure is at its highest. Daily life further requires ongoing trade-offs between competing goals: social interaction, physical activity, rest and sleep, and many individuals also seek periods of alone time.^12^^,^^13^ These multifaceted needs illustrate the necessity of a temporal structure that allocates different goals to different times of the day. Indeed, in most species, sleeping, feeding, and activity occur at specific times within the 24 h cycle, albeit with variations in timing between individuals.^14^^,^^15^^,^^16^^,^^17^ When motivation fails to align with time-of-day demands, counterproductive choices and actions can result. For example, excess social motivation in the evening can interfere with sleep,^18^ and low motivation to be active in the morning may make it difficult to get out of bed. Despite the necessity for a temporal structure, motivation has typically been studied as a relatively stable trait or, when treated as dynamic, as fluctuating from day-to-day.^19^^,^^20^ Recent work shows that day-to-day fluctuation in state motivation influences effort-based decision-making, with individuals exerting more effort and showing greater sensitivity to reward when feeling more motivated than usual.^19^ This work also showed that individuals high in apathy were especially dependent on momentary motivational states, suggesting that motivational impairments may reflect heightened state dependence rather than uniformly low motivation, but leaves open the question of whether motivation follows intra-day patterns, and whether individuals differ in these dynamics. If motivation varies within a single day, then the timing of motivational peaks and troughs could have direct consequences for self-regulation, mental health, and everyday functioning.

Laboratory studies provide evidence that motivational processes are modulated by time-of-day,^21^ but findings remain mixed in terms of the timing of peaks and troughs. Some studies report early afternoon peaks in risky decision-making, affective responses to positive images, and striatal response to monetary reward,^22^^,^^23^ whereas others find lows in reward learning or putamen activation to reward at similar times.^24^^,^^25^ Other work shows greater striatal responses to visual food stimuli in the morning compared with the evening^26^ and some studies find no diurnal differences in reward learning.^22^^,^^27^ Time-of-day variation has also been observed in reward-associated behaviors such as drug-craving for nicotine and heroin^28^^,^^29^ and in subjective liking and wanting, which tend to peak in the early evening.^30^ Together, these laboratory studies suggest that motivational processes are time-sensitive, but leave unresolved their timing, shape, and interindividual variability, as well as their relevance for everyday behaviors outside the laboratory. This limitation is compounded by the fact that most studies rely on sparse temporal sampling with only two or three measurement time points.

Understanding how subjective motivation is organized across the day is clinically and behaviorally relevant, as it may reveal when dysregulation is most likely and when support may be most effective. Self-reported momentary motivation offers direct access to individuals’ lived experience of wanting, effort, and goal prioritization. When assessed using ecological momentary assessment, these in-the-moment reports minimize retrospective bias by sampling motivation and behavior close in time and in naturalistic settings.^31^ A challenge is that individual differences in motivation can be substantial. Unsupervised clustering methods address this by classifying individuals into more coherent subgroups based on behavioral, cognitive, or psychiatric features. Unsupervised approaches, which operate without access to labels, can reveal subgroups that might otherwise remain hidden. Such techniques have identified subtypes of depression that predict treatment responses or latent patterns that signal vulnerability to adverse clinical outcomes,^32^^,^^33^ making them well-suited for exploring heterogeneity, identifying novel phenotypic profiles, and generating testable hypotheses for future research.

In the present study, we examined how everyday motivation unfolds across the day and how this temporal organization differs between individuals. Participants repeatedly reported their momentary motivation (“right now”) for four common states (resting, being alone, socializing, and exercising), providing a direct measure of subjective motivational states as they are experienced in daily life. Participants also reported whether they had engaged in behaviors in the preceding hours, allowing us to examine how fluctuations in motivation predict subsequent behavior. To capture trait-like individual differences in motivational functioning, we administered measures of depression, mania, inattention, hyperactivity/impulsivity, and apathy (cognitive, behavioral, and emotional subdomains) as prototypical indices of motivational dimensions. These measures capture a range of emotional and cognitive factors that shape how individuals initiate, sustain, and regulate behaviors, such as goal-directed motivation, behavioral activation, and sensitivity to reward, each of which is implicated in regulation of everyday behavior.^4^^,^^34^^,^^35^^,^^36^ We pursued four aims: (1) to identify motivational subgroups based on psychiatric traits commonly associated with altered motivation; (2) to characterize diurnal patterns of momentary motivation for everyday behaviors (resting, being alone, socializing, and exercising) and assess whether these patterns differ between motivational subgroups; (3) to examine diurnal patterns in everyday behaviors (spending time outside, exercising, socializing, and consuming caffeine), assessed as self-reported engagement in the past hours; (4) to determine how strongly motivation translates into behavior. By combining the measurement of stable individual differences (symptoms in the past weeks) with high-frequency assessment of momentary states across the day (“right now”), this study tests how everyday motivation is temporally organized within the day, how these patterns differ between individuals, and when motivation is most likely to drive behavior.

## Results

### Aim 1: Identify motivation clusters

**Figure 1:**
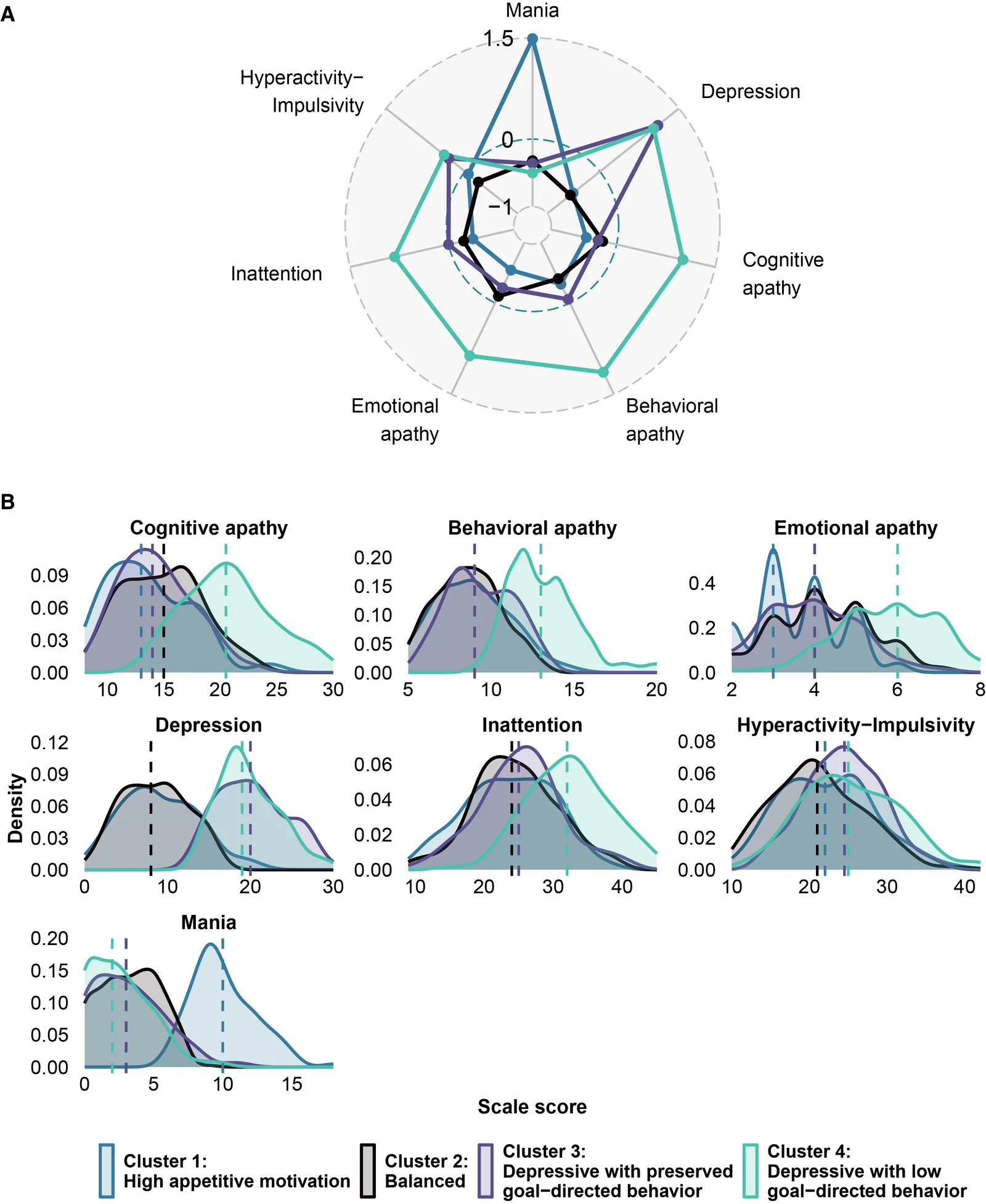
Characterization of motivation clusters (A) Radar plot showing the standardized mean scale scores for each cluster. (B) Density plots showing the distribution of raw scale scores within each cluster; dashed lines indicate median values. Cluster 1: *n* = 111; cluster 2: *n* = 253; cluster 3: *n* = 60; and cluster 4: *n* = 114.

Using Gaussian finite mixture modeling, we identified four clusters using 538 participants (424 women, 112 men, 2 non-binary; *M* age = 32.3 years, SD = 9.5, range 18–77) (see Figure 1). Cluster 1 (high-appetitive motivation; *n* = 111, 20.6%) was characterized by individuals with high levels of mania symptoms, low apathy, depression and inattention, and average hyperactivity/impulsivity. Cluster 2 (balanced; *n* = 253, 47.0%) showed symptom levels at or below the sample average across all measures. Cluster 3 (depressive with preserved goal-directed behavior; *n* = 60, 11.2%) was characterized by elevated depression symptom levels alongside relatively preserved cognitive and emotional goal-directed behavior (i.e., low levels of cognitive and emotional apathy, and average behavioral apathy and inattention), alongside moderate hyperactivity/impulsivity and low mania. Cluster 4 (depressive with low goal-directed behavior; *n* = 114, 21.2%) was marked by high apathy scores across cognitive, behavioral and emotional domains, elevated depression and inattention, moderate hyperactivity/impulsivity, and the lowest mania scores, reflecting broad motivational impairments.

For the remainder of the text, we will refer to the clusters as high motivation, balanced, depressive-preserved motivation, and depressive-low motivation, respectively.

Posterior classification probabilities indicated good assignment certainty across clusters, with mean posterior probabilities of 86.7% (SD = 16.3%) for cluster 1, 87.5% (SD = 15.6%) for cluster 2, 78.5% (SD = 18.1%) for cluster 3, and 80.8% (SD = 17.5%) for cluster 4.

There were modest deviations in the multivariate normality diagnostics, primarily related to skewness in two clusters (clusters 2 and 4) (see the supplemental information for sensitivity analyses). Compared with the GMM solution, the rank-based hierarchical clustering showed less separation between depressive profiles (clusters 3 and 4), suggesting more continuous variation in depressive symptom severity. Clusters 1 (high motivation) and 2 (balanced) were largely preserved, and diurnal motivation patterns were similar across methods. These findings indicate that clustering results are not fully dependent on distributional assumptions, while also suggesting that the cluster features vary along continuous dimensions and do not form sharply delineated subgroups.

### Aim 2: Time-of-day patterns of momentary motivation for everyday behaviors

We next asked whether motivation for everyday behaviors follows a diurnal pattern. Generalized additive mixed-effects models (GAMMs) showed nonlinear diurnal variation in all four motivational domains (Figure S7). Across participants, motivation to rest (edf = 5.36, F = 80.2, *p* < 0.001) and be alone (edf = 4.41, F = 24.2, *p* < 0.001) outweighed the motivation to socialize (edf = 4.57, F = 60.2, *p* < 0.001) and exercise (edf = 3.97, F = 106.5, *p* < 0.001) across the day. Motivation to rest and exercise and motivation to be alone and socialize showed paired diurnal profiles, although these relationships were not mirrors of each other. For example, the shapes of the curves were asymmetric, i.e., motivation to rest decreased from 8:00 to 12:00 and steeply increased thereafter, whereas motivation to exercise was flatter across the day. Moreover, low motivation to exercise does not necessarily imply a desire to rest, and low motivation to socialize does not necessarily reflect a wish to be alone, as shown by variable within-person correlations between individuals (see Supplemental Results “Within-person correlations of state motivations”). Specifically, motivation to rest was high in the early morning (∼08:00), declined until mid-day, and then rose steadily until late in the evening. Motivation to be alone was relatively stable between ∼08:00 and 19:00, followed by a pronounced evening increase. Motivation to socialize was highest ∼12:00-16:00 and declined thereafter. Motivation to exercise was highest in the early part of the day (∼08:00-13:00) and gradually decreased across the remainder of the day.

### Cluster differences in momentary motivation

**Figure 2:**
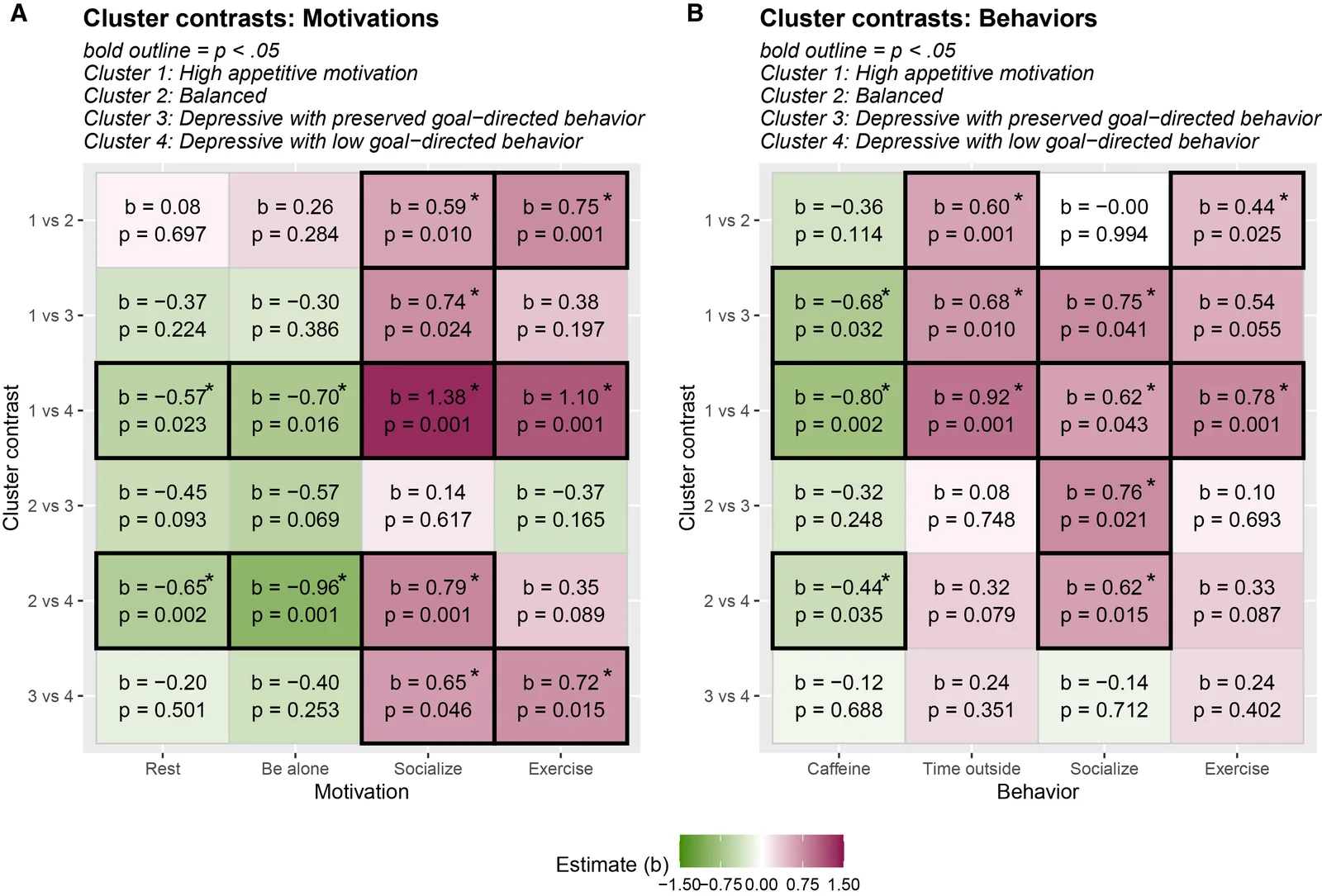
Cluster contrasts in motivations and behaviors Tile color represents the magnitude of between-cluster differences on the original scale (0–8). For caffeine, contrasts are expressed as log-odds differences; for example, cluster 1 shows half the odds of caffeine consumption compared with cluster 3 (OR = 0.51). Bold tile outlines indicate statistically significant contrasts (*p* < 0.05). Cluster labels are as follows: (1) high-appetitive motivation; (2) balanced; (3) depressive with preserved goal-directed behavior; and (4) depressive with low goal-directed behavior.

**Figure 3:**
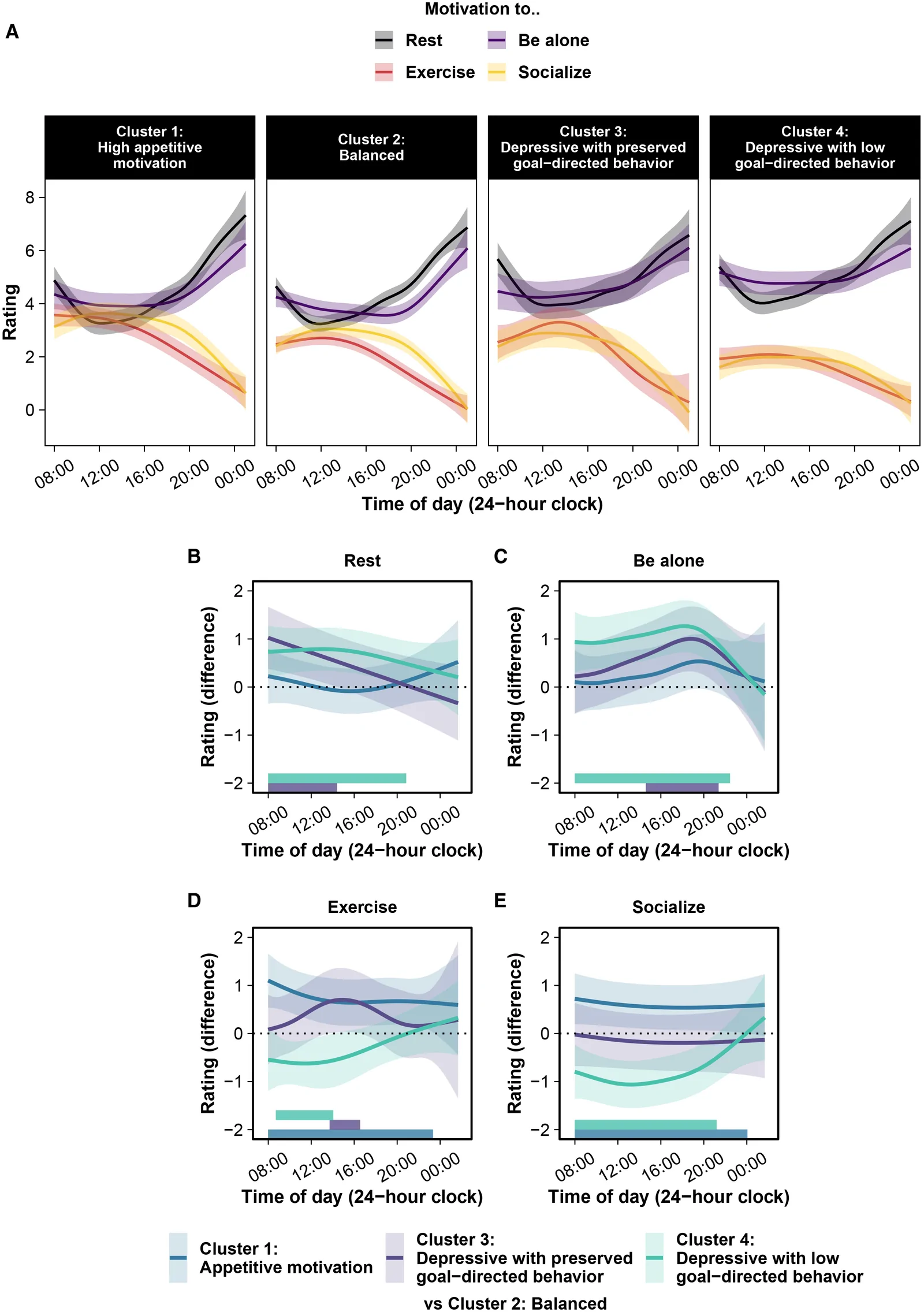
Time-of-day patterns of motivational states separated by cluster (A) Time-of-day patterns in motivation to rest, be alone, exercise, and socialize, stratified by motivation cluster. The *y* axis represents the item ratings (scale range: 0 “not at all” to 8 “very much/all the time”); error bands represent 95% pointwise confidence intervals (CIs). (B–E) Cluster difference smooths comparing each cluster (clusters 1, 3, and 4) to the balanced cluster (cluster 2). Positive values indicate higher motivation levels in the comparison cluster relative to the balanced cluster at that time-of-day. Error bands represent 95% CI of the difference. Colored horizontal bars below each curve denote time periods during which differences are statistically significant.

Full GAMM results are reported in Tables S3–S6 and visualized in Figures 2A and 3.

#### Cluster differences

Across clusters, momentary motivation levels differed after accounting for time-of-day and individual random effects (Figure 2A). The high motivation and balanced clusters reported lower motivation to rest and be alone than the depressive-low motivation cluster. Motivation to socialize and exercise was highest in the high-motivation cluster, exceeding all other clusters except that exercise motivation did not differ from the depressive-preserved motivation cluster. The depressive-low motivation cluster reported the lowest motivation to socialize and exercise.

#### Time-of-day cluster differences

Although the overall diurnal shapes of the motivation patterns were comparable across clusters, differences emerged in both level and timing. The depressive low-motivation cluster showed stronger motivation to rest and be alone than the reference (balanced cluster), particularly earlier in the day (Figures 3B and 3C), alongside lower motivation to exercise and socialize (Figures 3D and 3E), which showed attenuated diurnal variability (Figure 3A). The depressive-preserved motivation cluster likewise showed stronger motivation to rest in the morning (∼08:00–14:00) and to be alone in the late afternoon and evening (∼14:30–21:00). In contrast, the high-motivation cluster showed stronger motivation to exercise and socialize throughout the day, but most prominently earlier in the day (Figures 3D and 3E).

Clusters also differed in the relative strength of specific motivations across the day. In the high motivation and balanced cluster, motivation to socialize exceeded motivation to exercise from the afternoon, whereas in both depressive clusters, these motivations showed less separation. In the depressive-low motivation cluster, motivation to rest and be alone consistently outweighed the motivation to socialize and exercise, creating a pronounced gap between these opposing motivations. In the high-motivation cluster, the four motivations overlapped between the late morning and afternoon (∼10:00–15:00).

### Aim 3: Behavioral differences between motivational clusters

**Figure 4:**
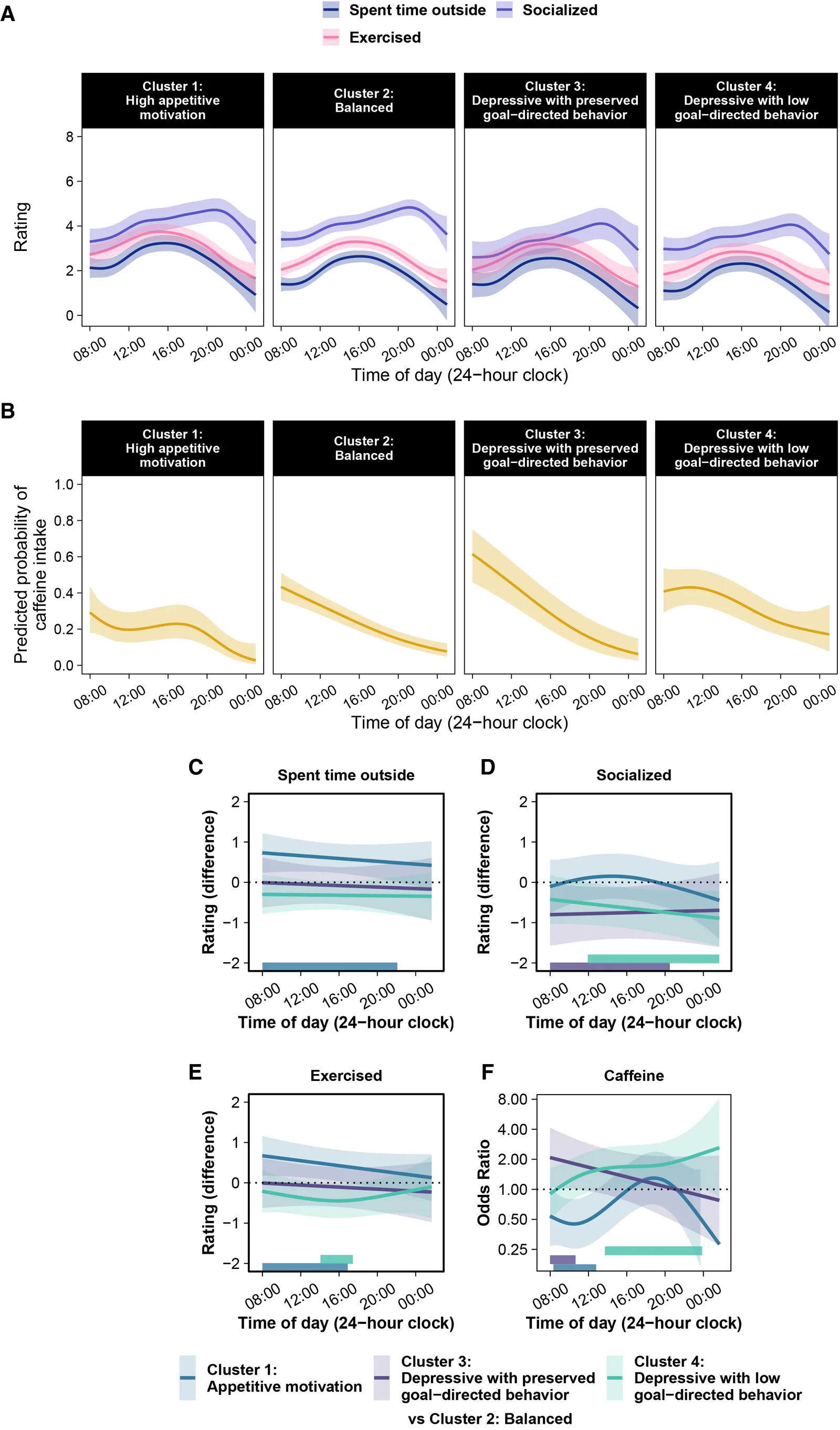
Time-of-day patterns of behavior separated by cluster (A and B) Time-of-day patterns of engaging in time spending time outside, socializing, exercising, and caffeine consumption, stratified by motivation cluster. Continuous outcomes (A) are shown on the original response scale (0 “not at all” to 8 “very much/all the time”); (B) caffeine consumption is shown as predicted probabilities for binary responses (yes/no); error bands represent 95% pointwise CIs. (C–F) Cluster difference smooths comparing each cluster (clusters 1, 3, and 4) to the balanced cluster (cluster 2). Positive values indicate more engagement in the respective behavior in the comparison cluster relative to the balanced cluster at that time of day. For (E), values above 1 indicate higher odds of caffeine consumption relative to the balanced cluster, and values below 1 indicate lower odds. Error bands represent 95% CI of the difference. Colored horizontal bars below each curve denote time periods during which differences are statistically significant.

Full GAMM results are reported in Tables S7–S10 and visualized in Figures 2B and 4.

#### Cluster differences

The high-motivation cluster reported less caffeine consumption, socialized more, and exercised more than the depressive clusters (except for exercising compared with the depressive-preserved motivation cluster), spent more time outside than all other clusters, and exercised more than the balanced and depressive-low motivation cluster. The balanced cluster also spent more time socializing than the depressive clusters. No significant behavioral differences were observed between the depressive-preserved motivation and depressive-low motivation cluster (see Figure 2).

#### Time-of-day cluster differences

The overall diurnal shapes of engaging in everyday behaviors were similar across clusters, with cluster-specific differences in the strength. Compared with the balanced cluster, the high-motivation cluster spent more time outside (Figure 4C) and engaged more in exercise (Figure 4E) earlier in the day. Both depressive clusters socialized less than the balanced cluster in the middle of the day, with the depressive-preserved motivation cluster from ∼08:00 to 20:00 and the depressive-low motivation cluster showing prolonged reductions into the evening (∼12:00-late) (Figure 4D). The high-motivation cluster reported lower morning caffeine consumption (∼08:15–12:30), whereas the depressive-preserved (∼08:00–10:00) and depressive-low motivation cluster showed higher caffeine consumption from the afternoon to evening (∼14:00–00:00) (Figure 4F).

### Aim 4: Momentary motivation predicts subsequent behavior

We next examined whether within-person fluctuations in motivation predicted engagement in subsequent behaviors, that is, whether momentary deviations in motivation from an individual’s mean level translated into corresponding changes in behavior at the next time point.

On average, exercise behavior was most strongly predicted by prior exercise motivation (b = 0.29, 95% confidence interval [CI] [0.23, 0.34], *p* < 0.001). Exercise behavior was also more likely following stronger motivation to socialize (b = 0.19, 95% CI [0.13, 0.25], *p* < 0.001) and less likely following stronger motivation to rest (b = −0.20, 95% CI [−0.25, −0.16], *p* < 0.001) and to be alone (b = −0.14, 95% CI [−0.19, −0.09], *p* < 0.001) (see Figure S8). On average, socializing was positively predicted by prior social motivation (b = 0.24, 95% CI [0.18, 0.31], *p* < 0.001), and was less likely following strong motivation to be alone (b = −0.24, 95% CI [−0.30, −0.19], *p* < 0.001) and to rest (b = −0.12, 95% CI [−0.17, −0.07], *p* < 0.001). Motivation to exercise did not significantly predict socializing behavior (b = 0.06, 95% CI [−0.00, 0.12], *p* = 0.066). On average, time spent outside was predicted by motivation to exercise (b = 0.30, 95% CI [0.24, 0.36], *p* < 0.001) and socialize (b = 0.27, 95% CI [0.21, 0.34], *p* < 0.001), as well as by lower motivation to rest (b = −0.22, 95% CI [−0.27, −0.17], *p* < 0.001), and to be alone (b = −0.16, 95% CI [−0.22, −0.11], *p* < 0.001). On average, caffeine consumption was slightly higher when exercise motivation was higher (odds ratio [OR] = 1.14, 95% CI [1.05, 1.24], *p* = 0.002). No other motivation level predicted caffeine intake. Full results are reported in the Supplementary Results and Figure S8.

#### Time-of-day modulation of within-subject motivation-behavior coupling

Finally, we examined whether the strength of the within-person motivation-behavior coupling varied across the day and between clusters. Analyses focused on behavior-specific motivations (social motivation predicting socializing and exercise motivation predicting exercising).

**Figure 5:**
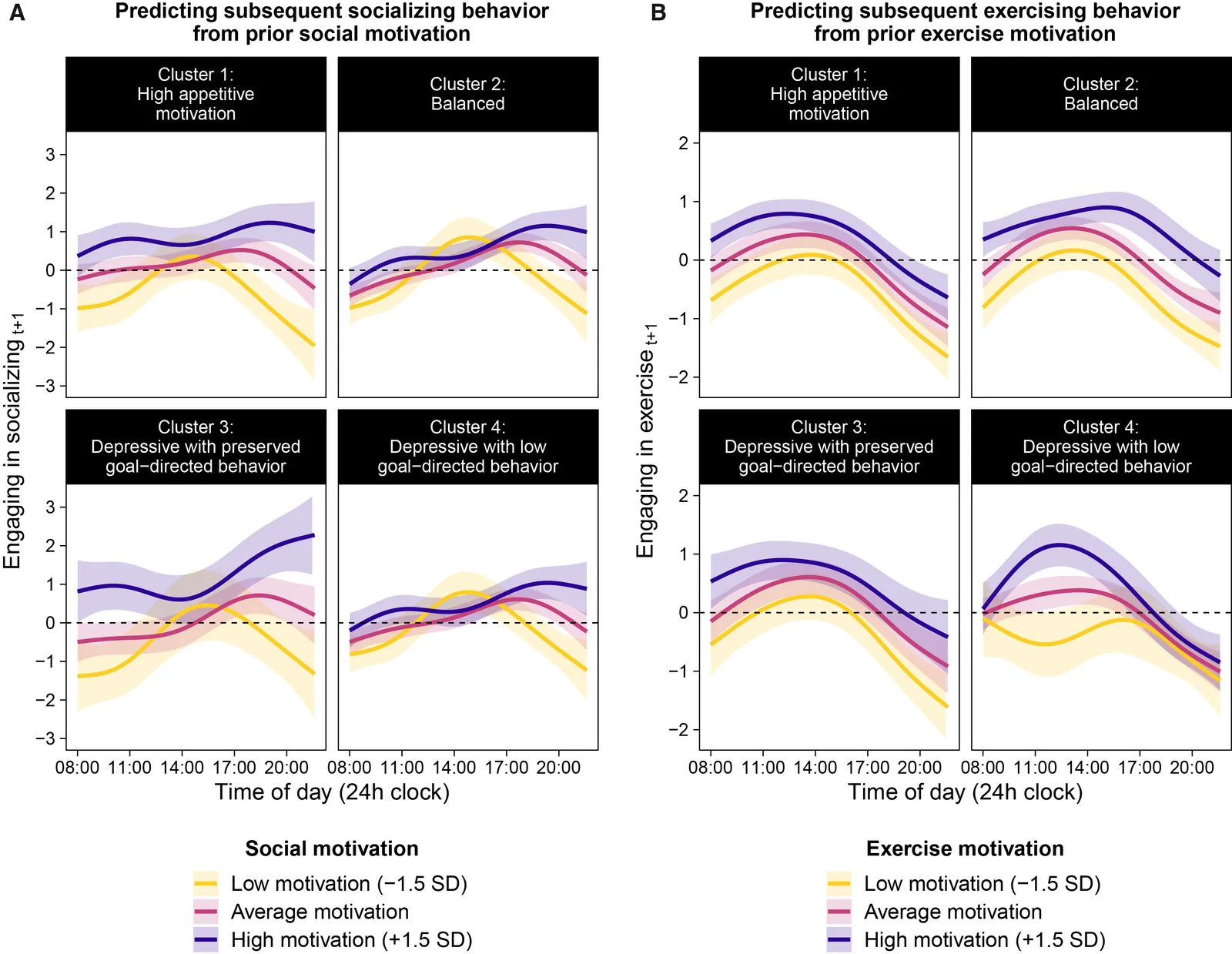
Time-of-day modulation of within-person motivation-behavior coupling across motivational clusters (A) Predicted deviations in subsequent socializing behavior (*t*_+1_) as a function of momentary social motivation (*t*) and time-of-day, shown separately for each motivational cluster. (B) Predicted deviations in subsequent exercising behavior (*t*_+1_) as a function of momentary exercise motivation (*t*) and time-of-day, shown separately for each motivational cluster. Lines represent model-predicted outcomes for within-person motivational levels that are lower than usual (−1.5 SD), average (mean), or higher than usual (+1.5 SD). Error bands represent 95% pointwise CIs. All outcomes are person-centered, such that values reflect deviations from an individual’s typical motivation/behavior. Motivation is modeled as a continuous predictor; categorical motivation levels are shown for visualization purposes only. The model answers “If at time *t*, a person’s motivation deviates from their average level, how is their behavior at time *t*_+1_ expected to change?”

The way social motivation translated into social behavior depended on time-of-day (time-of-day × social motivation: edf = 6.76, F = 1.82, *p* < 0.001). As shown in Figure 5A, both earlier and later in the day, higher-than-usual social motivation predicted increased socializing at *t*_+1_ and lower-than-usual motivation predicted reduced socializing at *t*_+1_. Cluster-specific modulation of this time-of-day × social motivation interaction was not supported. For exercising behavior, the association with exercise motivation was more equal across the day, but clusters differed in the timing of this coupling: in the depressive-low motivation cluster, exercise motivation more strongly predicted subsequent exercising earlier in the day (∼10:00–14:00) (edf = 1.74, F = 0.63, *p* = 0.027). In the balanced cluster, this coupling was somewhat stronger in the late afternoon (∼17:00) (edf = 1.00, F = 0.37, *p* = 0.031), as shown in Figure 5B.

### Cluster differences in chronotype

As an exploratory analysis, we examined whether chronotype is associated with the probability of belonging to a motivational cluster. Individuals with stronger morningness (i.e., higher chronotype scores) had a higher probability of belonging to the high-motivation cluster (OR = 1.05, 95% CI [1.02, 1.09], *p* = 0.001) and a lower probability of belonging to the depressive-low motivation cluster (OR = 0.92, 95% CI [0.89, 0.95], *p* < 0.001), both relative to the reference (balanced) cluster (Figure S9). Chronotype was not significantly associated with cluster membership of the depressive-preserved motivation cluster (OR = 0.97, 95% CI [0.93, 1.01], *p* = 0.116). These chronotype-cluster associations may help explain why the depressive-low motivation cluster displayed particularly lower activity-related motivation and elevated rest-oriented motivation in the earlier part of the day. This analysis involved a similar sample as used in^37^ where we tested relationships between psychiatric trait scores and chronotype.

## Discussion

This study demonstrates that everyday motivation is temporally structured, dominated by rest-oriented motives, and shaped by individual differences in trait motivation. We identified four motivational subgroups of trait-like psychiatric symptoms that tap into prototypical indices of altered motivation. These clusters—high-appetitive motivation, balanced, depressive with preserved goal-directed behavior, and depressive with low goal-directed behavior—predicted state levels of motivation for everyday behaviors, distinguishing their overall motivation levels and the relative weighting of recovery-oriented versus activity-oriented motivations. Momentary increases in social and exercise motivation predicted greater engagement in the corresponding behavior, however the strength of the motivation-behavior coupling varied by time-of-day, behavioral domain, and motivational phenotype. Social motivation was more likely to translate into social behavior in the morning and evening than at mid-day, potentially reflecting stronger contextual constraints during working hours. In contrast, the motivation-exercise link was relatively stable across the day, but individuals with greater trait-level deficits in goal-directed behavior were more sensitive to state exercise motivation between the late morning and afternoon. This extends prior work showing that willingness to exert effort is stronger driven by state motivation in individuals with higher trait apathy^19^ by demonstrating that trait low goal-directed behavior (apathy) influences the intensity of motivation for everyday behaviors, as well as the temporal conditions under which motivation is most likely translated into action.

Across the sample, motivation to rest and be alone outweighed the motivation to exercise and socialize at all times of day, suggesting a general prioritization of low-effort restorative states over more demanding activities. These findings highlight the centrality of energy conservation in daily life and align with cognitive and behavioral-economic models in which effort carries a subjective cost (i.e., energy-demanding actions are avoided when less demanding alternatives are available)^38^ (but also see Inzlicht et al.^39^ for a discussion that effort itself can be valued, with effort signaling meaning or reward). Motivation to exercise was strongest in the morning (08:00-12:00) and declined thereafter. A study by Budnick et al.,^40^ which tracked motivation to move and rest in 21 participants over eight days, reported a similar diurnal rhythm with declines in the afternoon but a later peak around 15:00, when motivation to rest was at its lowest. These findings point to a general daily rhythm in physical motivation, marked by a mid-day peak in the drive to move that coincides with reduced motivation to rest. However, these overall patterns mask individual differences. The cluster with low goal-directed behavior showed a wide motivational gap, defined as a large relative difference between activity-oriented and recovery-oriented motivations. Specifically, this cluster showed weak activity motivation and strong recovery motivation, coupled with attenuated diurnal variability in activity motivation. This gap potentially creates psychological or energetic barriers to engaging in socially or physically demanding behaviors. The high-appetitive-motivation cluster exhibited consistently higher motivation for activities compared with the balanced cluster, while motivation for rest did not differ between the clusters. This late morning to early afternoon overlap in motivation for being alone, resting, socializing, and exercising in the high-appetitive-motivation cluster suggests a period of co-activation across multiple motivational domains and may reflect greater motivational flexibility (ability to shift motivation between different behaviors depending on context). Alternatively, it could lead to internal tension when competing desires arise simultaneously. Future work could examine whether this co-occurrence of different motivations facilitates adaptive behavior (e.g., being ready to act on opportunities as they arise) or contributes to stress when choices must be made.

The motivational gap framework may be a useful concept for understanding why some individuals struggle to translate intentions into action and for guiding effective health interventions. The motivational gap is not a separate construct but an operational index of how motivations are distributed at a given moment, i.e., the relative weighting of competing motivations. Understanding this balance may therefore be more informative than focusing on activity motivation alone, as it captures the trade-off inherent to daily motivation. Traditionally, behavioral activation programs often aim to increase motivation for specific behaviors, such as physical activity, through education or incentives.^41^ Yet, our findings motivate future research to test a complementary route in which interventions are timed to coincide with moments of the day when the motivational gap is smaller, such as around noon. Recalibrating rest-related motivation, for instance, by reframing rest through short restorative activities with active elements, could also be tested in future work as a potential way to shift this balance.

Clusters differed in their amount of time spent outside, socializing, and exercising, although differences were less strong than between-subject motivational differences, possibly reflecting environmental structures (e.g., commuting to school or work) overriding internal motivational states. Depressive clusters spent the least time outside, socializing, and exercising, but consumed more caffeine. Caffeine consumption was lower in the high-appetitive-motivation cluster than the depressive clusters, which may reflect a naturally higher baseline level of energy or arousal in the high-appetitive-motivation cluster. This contrasts with findings in clinical populations, where individuals with bipolar disorder have been reported to have higher serum caffeine levels compared with controls.^42^ Although the clinical implications of high caffeine levels are not well understood, acute increases in caffeine consumption have been proposed to precede the occurrence of manic symptoms in bipolar disorder.^43^ The higher consumption of caffeine in the depressive clusters is consistent with a “self-medication” account. Caffeine may be used to counteract low energy, fatigue, or side effects of medication.^44^ Some studies have linked greater caffeine consumption to lower depression symptoms, but findings remain inconclusive.^45^ It is unclear whether higher caffeine consumption in the current study reflects an attempt to manage symptoms, whether it is ineffective (or only partially effective), or whether other factors drive both caffeine consumption and depressive symptoms. The high-appetitive-motivation cluster also spent more time outside (compared with all other clusters) and more socializing (compared with the depressive clusters) and exercising (compared with balanced and depressive-low motivation clusters), which suggests greater behavioral activation and potentially a preference for stimulating environments. Together, these behavioral differences suggest that trait motivation shapes what people feel inclined to do, but also interacts with daily behavior and energy compensatory behaviors such as caffeine consumption.

The similarity in the general shape of diurnal motivation across clusters points to a shared regulatory process that coordinates the rest-activity cycle. An exception was the depressive-low motivation cluster, which showed reduced diurnal variability in motivation to exercise and socialize, consistent with a generalized blunting of goal-directed behavior. The circadian system likely plays a role in orchestrating these daily peaks and troughs in motivation, potentially aligning motivational rhythms with biologically relevant windows for exertion and recovery.^46^^,^^47^ Given the central role of dopamine in motivation and behavioral activation, this modulation may be partly mediated by circadian variations in dopaminergic signaling, which have been observed in animal models.^48^^,^^49^ For example, phasic dopamine responses are amplified during phases of the circadian cycle when reward-seeking would be advantageous (i.e., nighttime in mice).^49^ While the extent to which this applies to humans remains to be determined, disruptions to circadian functioning, such as altered rest-activity rhythms and delayed sleep, are common in depression and mania^50^^,^^51^ and may interfere with the synchronization and amplitude of motivational rhythms, thereby widening the motivational gap. Supporting this interpretation, our exploratory analyses showed that morningness was more prevalent in the high-appetitive-motivation cluster (with elevated trait mania) and eveningness was more prevalent in the depressive with low goal-directed behavior cluster. This pattern aligns with evidence linking delayed sleep-wake rhythms (eveningness) to mood disorders and advanced sleep-wake rhythms (morningness) to mania.^52^^,^^53^

To conclude, daily motivation follows rhythms that are dominated by rest-related motivations, but trait-like differences in motivation shape how strongly and when motivation translates into behavior. The *motivational gap*, the relative difference between multiple coexisting motivations, offers a dynamic framework for understanding when intended actions succeed or fail and provides time-sensitive intervention levers. Future work may test whether interventions, such as those promoting exercise, are more effective when they target multiple sides of this balance: strengthen approach motivations toward desired behaviors, temper the pull of low-effort/maladaptive alternatives, and exploit the timing when alignment is naturally most favorable.

### Limitations of the study

Several limitations should be considered when interpreting the current findings. First, data collection was conducted remotely. While this approach offers higher ecological validity than laboratory-based assessments of subjective motivation, it also introduced potential variability in environmental conditions that could not be controlled or verified directly. Second, an uneven gender distribution reduced statistical power to assess gender differences. Third, all data were collected on weekdays, limiting generalizability of the findings across the full week, as motivational states^19^ and behavioral opportunities often differ between weekdays and weekends. Fourth, situational constraints, such as work demands or physical limitations, are likely to shape both motivation and behavior. These contextual factors were not assessed in the EMA measures, limiting our ability to examine how situational opportunities or constraints may have influenced motivation and behavior. Future work could integrate contextual measures to assess how situational opportunities interact with motivational processes, as well as address the role of sleep. Finally, multivariate normality of the joint distributions within each cluster was not fully supported, and this motivates cautious interpretation. The clusters should be viewed as informative theory-driven approximations that summarize multivariate patterns in psychiatric dimensions rather than as definitive latent categories. This warrants validation in larger samples. Combining both subjective and objective behavioral measures and more days with temporally dense sampling will also be important to further refine models of motivation-action coupling.

## Resource availability

### Lead contact

Requests for further information should be directed to the lead contact, Leonie J.T. Balter (leonie.balter@ki.se).

### Materials availability

This study did not generate new unique reagents.

### Data and code availability

• The data supporting the current study are available at the Open Science Framework at https://doi.org/10.17605/OSF.IO/JQY7C.
• The original code is available at the Open Science Framework at https://doi.org/10.17605/OSF.IO/JQY7C.
• Any additional information required to analyze the data reported in this paper is available from the lead contact upon request.

## Acknowledgments

This work was supported by grants from Rut and Arvid Wolff 10.13039/100028926Memorial Foundation (no. FS-2021:0008, to L.J.T.B.). European Union’s 10.13039/501100007601Horizon 2020 research and innovation program under the Marie Skłodowska-Curie grant agreement (no. 101108620, to L.J.T.B.).

## Author contributions

J.A., conceptualization, methodology, interpretation, and writing – review and editing; R.C., conceptualization, interpretation, and writing – review and editing. L.J.T.B., conceptualization, methodology, software, investigation, formal analysis, interpretation, writing – original draft, writing – review and editing, visualization, project administration, and funding acquisition. All authors contributed to, reviewed, and approved the final version of this manuscript.

## Declaration of interests

The authors declare no competing interests.

## STAR★Methods

### Key resources table

**Table:** 

REAGENT or RESOURCE	SOURCE	IDENTIFIER
**Deposited data**		

Deposited data	This paper	OSF: https://doi.org/10.17605/OSF.IO/JQY7C
Analysis script	This paper	OSF: https://doi.org/10.17605/OSF.IO/JQY7C

**Software and algorithms**		

R version 4.5.1	The R Project	https://www.r-project.org/
RStudio version 2025.05.1 + 513	Posit	https://posit.co/downloads
mclust	Scrucca et al.	https://cran.r-project.org/web/packages/mclust/index.html
mgcv	Wood	https://cran.r-project.org/web/packages/mgcv/index.html
psych	Revelle	https://cran.r-project.org/web/packages/psych/index.html
MVN	Korkmaz et al.	https://cran.r-project.org/web/packages/MVN/index.html
gratia	Simpson	https://cran.r-project.org/web/packages/gratia/index.html

**Other**		

Prolific recruitment platform	Prolific	https://www.prolific.com/
Center for Epidemiologic Studies Depression Scale Revised Short Form (CESD-R 10)	Cole et al.^54^	https://doi.org/10.1037/1040-3590.16.4.360
Altman Self-Rating Mania Scale (ASRM)	Altman et al.^55^	https://doi.org/10.1016/S0006-3223(96)00548-3
Adult ADHD Self-Report Scale (ASRS)	Adler et al.^56^	https://doi.org/10.1080/10401230600801077
Apathy Evaluation Scale (AES)	Marin et al.^57^	https://doi.org/10.1016/0165-1781(91)90040-V

### Experimental model and study participant details

#### Study participants

Data from 538 participants (424 women, 112 men, 2 non-binary; *M* age = 32.3, *SD* = 9.5, range 18-77) were included in the latent cluster analysis conducted on baseline data (Day 1, see procedures). For the diurnal assessments conducted the following day, participants who completed at least two of the six timepoints were included in the diurnal analyses (*N* = 410, 320 women, 88 men, 2 non-binary) (*M* age = 32.2, *SD* = 9.0, range 18-63). The median number of completed timepoints was six. Participants were recruited through Prolific.co, an online platform for academic research. Eligibility criteria included residing in the United Kingdom, fluency in English, being 18 years or older, and a Prolific approval rate of ≥99% in previous participations were invited to participate. The latter means that at least 99% of the participant’s past submissions were deemed valid and approved by researchers in previous studies. Participants received £7.50 per hour, with an additional £5 bonus for completing all timepoints. The total time commitment for participants was about 2-2.5h. Data collection took place in 2021. The study was approved by the Swedish Ethical Review Authority (dnr: 2021-01695). All participants provided online informed consent prior to participation.

### Method details

#### Procedures

The study was conducted across two consecutive weekdays.

##### Day 1 (baseline session)

Between ∼09:00 and 21:00, participants completed self-report measures on demographics, medical diagnoses, psychiatric traits, sleep, and chronotype. Analyses focused on seven constructs: depression, mania, inattention, hyperactivity/impulsivity, and apathy (with cognitive, behavioral, and emotional subdomains). The rationale for including these constructs was that they together span psychiatric symptoms commonly associated with alterations in goal-directed motivation. Depression has been linked to reward-processing impairments, low energy, and withdrawal tendencies (hypo-motivation).^58^^,^^59^ Mania is characterized by heightened approach/activation (hyper-motivation). Inattention captures deficits in sustaining task goals and deploying control, and hyperactivity/impulsivity indexes motor activation and a tendency toward immediate actions. Apathy is a multidimensional syndrome indexing diminished initiation and maintenance of action and effort mobilization, with subdomains reflecting diminished initiation (behavioral), diminished goals (cognitive), and blunted emotional reactivity (emotional).^3^^,^^4^ This set of measures provides complementary coverage of partially dissociable components of everyday motivation.

##### Day 2 (diurnal assessments using ecological momentary assessments [EMA])

Participants completed six EMA timepoints across the day, spaced approximately three hours apart between ∼08:00 and 23:00. At each timepoint, they reported whether they had engaged in certain behaviors since the previous assessment, rated their momentary motivation, and completed a brief cognitive test battery (data are not reported here). For this study, eight ratings were analyzed: four motivations (motivation to rest, be alone, socialize, and exercise) and four behaviors (whether engaged in exercise, social interaction, time spent outside, and caffeine consumption). All EMA items were completed in a fixed order. Participants were instructed to refrain from taking naps during the study. No information about napping during the study was collected. All data were timestamped, and timestamps were used for the analyses. Further methodological details are available in the Supplementary Materials and in related publications using partially the same sample.^37^^,^^60^^,^^61^

#### Measures

Trait measures (symptoms in the past weeks) of depression, mania, inattention and hyperactivity/impulsivity, and behavioral, cognitive, and emotional apathy were completed on Day 1. On Day 2, state measures of motivation for daily life behaviors (“at this moment”) and engagement in behaviors since the previous EMA prompt (or “since this morning” for the first EMA of the day) were assessed repeatedly. The measures were completed in the order as presented below.

#### Psychiatric trait scales

##### Depression

The Center for Epidemiologic Studies Depression Scale Revised Short Form (CESD-R 10)^54^ is a 10-item scale designed to measure depressive symptoms in the general population. Items are rated on a 4-point scale from 0 (“rarely or none of the time”) to 3 (“almost all of the time”), with two items reverse scored. Total scores were computed by summing item ratings. The CESD-R 10 showed excellent internal consistency (Cronbach’s α = 0.88). Bifactor reliability indices indicated high reliability of the total score (ω_t_ = 0.91) with substantial variance accounted for by a general depression factor (ω_h_ = 0.74; Explained Common Variance [ECV] = 0.66).

##### Mania

Mania symptoms were measured using the 5-item Altman Self-Rating Mania Scale (ASRM),^55^ which assesses behaviors and thought patterns, such as increased self-esteem, reduced need for sleep, and heightened talkativeness relative to one’s usual state. Items are rated on a 5-point scale (0-4) and summed to yield a total score. Internal consistency was acceptable (α = 0.77). Bifactor reliability indices supported the reliability of the total score (ω_t_ = 0.78) with substantial saturation by a general mania factor (ω_h_ = 0.77). The ECV was not calculated because only a single factor was extracted.

##### Inattention and hyperactivity/impulsivity

Symptoms of inattention and hyperactivity/impulsivity were assessed using the Adult ADHD Self-Report Scale (ASRS).^56^ The ASRS consists of 18 items reflecting DSM-IV Criterion A symptoms of adult ADHD. The inattention subscale reflects difficulties with sustained focus, organization, and task completion, whereas the hyperactivity/impulsivity subscale represents restlessness, excessive movement, and impulsive decision-making. Items are rated on a 5-point frequency scale from 1 (“never”) to 5 (“very often”). The scale demonstrated high internal consistency (α = 0.88). Bifactor reliability indices indicated strong reliability of the total score (ω_t_ = 0.90) with moderate saturation by a general ADHD factor (ω_h_ = 0.50; ECV = 0.44), supporting a multidimensional structure.

##### Apathy

The Apathy Evaluation Scale (AES)^57^ measures behavioral, cognitive, and emotional concomitants of deficits in goal-directed behavior. This 18-item scale is scored on a 4-point scale, from 1 (“not at all”) to 4 (“a lot”), with three items reverse scored. Subscale and total scores were calculated by summing relevant items. The AES showed excellent internal consistency (α = 0.90). Bifactor reliability indices indicated high reliability of the total score (ω_t_ = 0.92) with saturation by a general apathy factor (ω_h_ = 0.84; ECV = 0.75), suggesting that most common variance is captured by a general factor, with more limited unique contributions of the subscales.

#### EMA items

##### Engagement in behaviors

Engagement in exercise, social interaction, time spent outside, and caffeine consumption was assessed using four items; “Have you been physically active?”; “Have you spent time with other people?”; “Have you spent time outside?”; “Have you consumed caffeinated products such as coffee or energy drinks?” respectively. The first three items were rated on a scale from “1 = Not at all” to “9 = Very much/All the time” and rescored to 0 to 8 for the analyses. Caffeine consumption was rated as a binary response (Yes or No).

##### Motivation for daily life behaviors

Motivation for resting, being alone, socializing, and exercising was assessed using four items: “How much would you like to rest?”; “How much would you like to be by yourself?”; “How much would you like to be with a group of friends?”; “How much would you like to work out/exercise?”, respectively. Items were rated on a scale from “1 = Not at all” to “9 = Very much/All the time” and rescored to 0 to 8 for analyses.

### Quantification and statistical analysis

#### Scale scoring and reliability assessment

For each questionnaire, internal consistency was evaluated using Cronbach’s alpha, omega total (ω_t_), omega hierarchical (ω_h_), and Explained Common Variance (ECV). The *omega* function of the psych R package^62^ was used to estimate reliability indices for the CESD-R 10, ASRM, ASRS, and AES. ω_total_ quantifies the proportion of total-score variance that is reliable (1-ω_total_ reflects random error). The ω_h_ is the proportion of total-score variance attributable to a single general factor in a bifactor model. Values of ≥ 0.70 are considered meaningful for a strong unidimensional scale. The ECV is a measure to judge the degree of unidimensionality by indexing the proportion of common variance in the total score accounted for by the general factor. A high ECV suggests the scale is more unidimensional.^63^^,^^64^

#### Identification of motivation clusters

Gaussian finite mixture modelling, as implemented in the Mclust R package,^65^ was used to identify motivation clusters. This model-based clustering technique assumes that data are a mixture of observations from different latent subpopulations, and tries to deduce information of underlying distributions. Each cluster is represented by a probability distribution model, and the goal is to estimate the parameters of these models to best fit the observed data. Mclust uses the expectation-maximization (EM) algorithm to estimate the parameters of the mixture model. This algorithm iteratively updates the cluster assignments and model parameters to maximize the likelihood of the observed data given the model. The number of clusters was guided by a combination of the fit statistic Bayesian Information Criterion (BIC), bootstrap Likelihood Ratio Testing (LRT), and theoretical considerations. The BIC balances model fit against complexity, favoring more parsimonious solutions for the same relative degree of fit. The LRT tests whether introducing an additional cluster yields a statistically significant improvement in model fit. Clustering was performed on trait-level measures indexing depression, mania, inattention, hyperactivity/impulsivity, and cognitive, behavioral, and emotional apathy. Multivariate distribution assumption of the joint distributions within each latent cluster was tested using Mardia’s test for multivariate normality using the MVN R package.^66^ Since multivariate normality was not fully supported for all clusters, we conducted rank-based hierarchical clustering as a sensitivity analysis.

#### Diurnal variation in motivation and behavior

Generalized additive mixed-effects models (GAMMs) were used to assess diurnal patterns in momentary motivation and behavior, using the mgcv R package.^67^ GAMM uses smooth functions to capture both linear and non-linear patterns, allowing for flexible representations of diurnal patterns. For each outcome, we fitted a model with a random effect for participant id s(id, bs = 're'), a smooth term for time-of-day (continuous) to capture the overall diurnal variation s(time of day, bs = 'tp'), and cluster as a categorical variable to assess parametric mean differences (cluster). To test whether the diurnal shapes differ by cluster, we used cluster-specific deviation smooths of time-of-day by cluster, giving a separate curve for each cluster with the thin plate regression spline s(time of day, by = cluster, bs = 'tp', m = 2). m = 2 applies a second-derivative penalty so that it penalizes curvature in the per-cluster curves. Cluster was modeled as a between-subjects factor and the resulting diurnal curves reflect average motivational and behavioral patterns across individuals within each cluster. The effective degrees of freedom (edf) describe the degree of nonlinearity of a smooth.^67^ An edf of ∼1 indicates an approximately linear deviation, and a larger edf indicates increasing non-linearity in the cluster-specific curve. Motivational and behavioral outcomes were modeled using a Gaussian family. Caffeine consumption (yes/no) was modeled using a binomial distribution with logit link. Missing data (7.7%, 186 out of 2413 timepoints) were handled via listwise deletion. Difference smooths were extracted using the Gratia R package.

#### Predicting behavior from prior motivation

To test whether momentary motivation predicts subsequent behavior, motivation reported at assessment *t* was used to predict behavioral engagement at assessment *t* + *1*. All momentary motivation and behavior variables were person-mean centered to reflect within-person variations. This strategy partitions out between-person differences, so that the effects reflect within-person variation. A one-unit increase represents higher motivation or behavioral engagement relative to the individual’s mean. Associations between motivation and subsequent behavior averaged across the day were first estimated using linear models. To examine whether the strength of motivation-behavior coupling differed between clusters and across the day, we fitted GAMs with a random intercept for participant to account for repeated assessments. Time-varying associations between within-person motivation and behavior were modeled using smooth terms for motivation s(motivation, bs = ‘tp'), and time-of-day s(time of day, bs = ‘tp'), as well as a tensor-product smooth of time-of-day and motivation ti(time of day, motivation, bs = ‘tp'). To test for cluster-specific modulation of this coupling, we included a cluster-specific tensor-product interaction for time of day, motivation, and cluster ti(time of day, motivation, by = cluster, bs = ‘tp'), allowing the shape and timing of effects to vary by cluster. All corresponding lower-order terms were included to maintain a hierarchical model structure. Double penalty shrinkage was used to stabilize estimation in the presence of multiple smooth terms and interactions, allowing unsupported smooth terms to be penalized toward zero. Basis dimensions were set conservatively to support stable estimation of cluster-specific and interaction smooths. For all GAM models, smoothing parameters were selected automatically using restricted maximum likelihood (REML).
