# Supplementary material for: Time-of-day variation in motivation for everyday behaviors: A cluster-based approach to understanding individual differences
Source: iScience. 2026 Sep 7;29(9):117366. doi: 10.1016/j.isci.2026.117366 (PMC13577883; doi:10.1016/j.isci.2026.117366)
Supplement: Supplementary file 1 [file mmc1.pdf]

iScience, Volume *29*

Supplemental information

Time-of-day variation in motivation
for everyday behaviors: A cluster-based approach
to understanding individual differences

John Axelsson, Roshan Cools, and Leonie J.T. Balter

Supplementary Information

Diurnal variation in motivation for everyday behaviors: A cluster-based approach to understanding individual differences

John Axelsson, Roshan Cools, & Leonie JT Balter

Table of Contents

Supplementary Methods ***3***
1.1 Recruitment 3
1.2 Study design 3
1.2.1 Dropout and exclusion 3
1.3 Measures 4
1.3.1 Inattention 4
Supplementary Results ***5***
2.1 Autocorrelation sensitivity analysis 5
2.2 Multivariate and univariate normality assessment 5
2.3 Within-person correlations of state motivations 7
Supplementary Figures ***8***
Fig. S1. Correlation matrix of the psychiatric scales 8
Fig. S2. Frequency distributions of motivation and behavior items 9
Fig. S3. Frequency plots of the psychiatric scale scores 10
Fig. S4. Model selection indices for determining the optimal number of latent clusters 11
Fig. S5. Posterior classification probabilities for each participant across clusters 12
Fig. S6. Bootstrap distributions of GMM cluster means (parameter stability) 13
Fig. S7. Time-of-day patterns of motivation 14
Fig. S8. Predicting behavior from prior motivation 15
Fig. S9. Association between cluster membership and chronotype 16
Fig. S10. Normal Q–Q plots of trait variables by latent cluster 17
Fig. S11. Rank-distance clustering and corresponding diurnal motivation patterns 18
Fig. S12. Distribution of within-person correlations of state motivations 19
Supplementary Tables ***20***
Table S1. Age and gender distribution in the full sample (N = 538) 20
Table S2. Age and gender distribution in the EMA subsample (N = 410) 21
Table S3. Generalized additive mixed model (GAMM) results predicting Wanting to rest 22
Table S4. GAMM results predicting Wanting to be alone 22
Table S5. GAMM results predicting Wanting to socialize 22
Table S6. GAMM results predicting Wanting to exercise 23
Table S7. GAMM results predicting Time outside 24
Table S8. GAMM results predicting Socializing behavior 24
Table S9. GAMM results predicting Exercising 25
Table S10. GAMM results predicting Caffeine intake 25
Table S11. GAMM results predicting Socializing from motivation 26
Table S12. GAMM results predicting Exercising from Exercise motivation 26
Table S13. Percentage of missing EMA observations at each timepoint by cluster and data collection ID 28
Table S14. Comparison of smooth terms between primary GAMMs and AR(1) sensitivity models for motivation EMA items 29
Table S15. Comparison of smooth terms between primary GAMMs and AR(1) sensitivity models for behavioral EMA items 30
Table S16. Multivariate normality test 31

Supplementary Methods

This section provides additional details on participant recruitment, study procedures, dropout and exclusion criteria, and measures.

1.1 Recruitment

Sixty-five participants were recruited to assess feasibility and dropout rates. Participants in this initial batch completed Day 1 (baseline session) and five timepoints, separated by approximately four hours, between ~08:00-00:00. The subsequent data collections included six timepoints, separated by approximately three hours between ~08:00-00:00.

1.2 Study design

Data collection for this study occurred on weekdays only. Participants were divided into two groups for Day 2 assessments. All participants were instructed to start the first EMA timepoint on Day 2 within the time window of 08:00-09:00. This time of the first assessment was self-chosen to reduce potential interference with habitual sleep duration/wake time. This morning timepoint was followed by five timepoints with start times that were separated by three hours. Group 1 was instructed to start the subsequent timepoints at 10:00, 13:00, 16:00, 19:00, and 22:00, and group 2 was instructed to start the subsequent timepoints at 11:00, 14:00, 17:00, 20:00, and 23:00. The one-hour shift between the groups provides data for more timepoints across the day. All data were timestamped, and the timestamp was used for the analysis. Participants were encouraged to start each completion at the instructed time; however, they could complete each respective timepoint also after the start times in order to maximize the number of available data points.

1.2.1 Dropout and exclusion

Participants were included in the baseline analyses if they completed all measures required for the current analyses, defined as completion of all measures up to and including the Apathy Evaluation Scale. This resulted in a final baseline sample of 538 participants. Of these, 410 participants contributed valid EMA data on the following day. Participants were excluded from

the EMA analyses if they completed a single EMA assessment (n = 27). The median number of completed EMA timepoints was six. Missing EMA timepoints are reported separately by cluster and data collection wave in Table S13, where data collection ID 0 refers to the 65 participants scheduled for five timepoints between 8:00 and 00:00 and data collection ID 1 refers to the six timepoint protocol (8:00-23:00). Later timepoints had more missing data.

1.3 Measures

Psychiatric scales that asked for symptoms over the past week or two weeks were adapted to ask for symptoms over the past month, applying to the CESD-R 10 (depression) and ASRMS (mania symptoms).

1.3.1 Inattention

To control for possible inattention during data collection, two attention checks and one honesty check were included in the baseline session (Oppenheimer et al., 2009): “Please rate the response alternative 'agree’ for this question”; “Please answer 100”; “Have you been completely honest in your answers?”. Baseline data of participants who failed more than one of these checks (*n* = 6) were excluded.

Supplementary Results

This section presents additional analyses evaluating model robustness, distributional assumptions, and within-person associations between momentary motivational states.

2.1 Autocorrelation sensitivity analysis

To evaluate whether residual autocorrelation influenced the reported diurnal and cluster-specific effects, we refitted the full models using an AR(1) residual correlation structure, which accounts for correlations between adjacent observations. The autoregressive parameter (ρ) was approximated using the lag-1 correlation of residuals from the primary models and was then supplied when refitting the models. Time series were restarted at the beginning of each participant's observations. Inclusion of the AR(1) structure did not materially alter the results. Estimated smooth terms remained highly similar in shape and magnitude, and statistical inferences were unchanged across models. AR(1) models yielded modest reductions in the effective degrees of freedom for some terms (e.g., the participant-level random intercept), without affecting conclusions. These findings suggest that the reported diurnal patterns are unlikely to be attributable to residual autocorrelation. Models were compared with respect to estimated smooth shapes and effective degrees of freedom and are presented in Table S14 and Table S15.

2.2 Multivariate and univariate normality assessment

To evaluate distributional assumptions underlying the multivariate GMM, multivariate normality was assessed within each latent cluster using Mardia's tests of skewness and kurtosis, as implemented in the MVN R package (Korkmaz et al., 2014). For two clusters (Cluster 1 and Cluster 3), neither Mardia's skewness nor kurtosis tests indicated a significant deviation from multivariate normality. For Cluster 2 and Cluster 4, Mardia's skewness test indicated deviations from multivariate normality, whereas kurtosis was not significant in any cluster. These findings suggest some asymmetry in the joint distributions (Table S16). To further examine distributional

properties, we inspected univariate normality within each cluster for all measures used in the multivariate GMM. Q-Q plots are shown in Fig. S10. Across clusters, most variables broadly approximated a Gaussian distribution, although departures from normality were evident for some measures, primarily in the distributional tails. Emotional apathy showed a stepwise pattern consistent with its restricted response range (2-8). Most patterns were observed across clusters and not cluster-specific.

These results indicate that departures from normality were present, primarily related to skewness. As a sensitivity analysis, we therefore repeated the cluster analysis using a rank-based hierarchical approach that does not rely on Gaussian distributional assumptions. This alternative approach yielded a broadly similar pattern of clusters and GAMM-derived diurnal motivational patterns (Fig. S11). The primary difference concerned the separation of the depressive profiles represented by Cluster 3 and 4. Under the rank-based approach, Cluster 3 was characterized by moderate-to-low goal-directed behavior with average levels of depression, rather than the more sharply elevated depression with preserved goal-directed behavior as observed in the GMM-derived cluster. Consequently, the rank-based approach produced less separation between the depressive profiles, suggesting that, when distributional assumptions are relaxed, depressive symptom severity may vary more continuously between individuals rather than forming strictly separated depressive subgroups. In contrast, Cluster 1 (high appetitive motivation) and Cluster 2 (balanced) were largely preserved across methods. The diurnal patterns of momentary motivation to rest, be alone, exercise, and socialize showed similar shapes across clustering methods, with the level of goal-directed behavior driving the separation between recovery-oriented and activity-oriented motivations. This is consistent with the stronger separation between recovery-oriented versus activity-oriented motivations observed in the rank-based Cluster 3 compared with the GMM-derived Cluster 3, as the rank-based Cluster 3 was characterized by more pronounced low goal-directed behavior.

Considering the modest distributional deviations and the partial convergence across clustering methods, these findings suggest that the observed clustering structure is not fully dependent on distributional assumptions. At the same time, the results indicate that the

identified clusters should be interpreted primarily as reflecting variation along continuous dimensions, rather than as sharply delineated discrete subgroups. The GMM cluster profiles are further consistent with theoretically interpretable profiles of motivational and symptom dimensions, supporting their use as a descriptive framework for characterizing individual differences in motivation for daily life behaviors.

2.3 Within-person correlations of state motivations

To examine whether state motivations reflect bipolar opposites at the within-person level, we quantified the coupling between state motivation items separately for each individual. We focused on motivation to exercise versus motivation to rest, and motivation to socialize versus motivation to be alone. Momentary motivation ratings were person-mean centered to remove between-person differences in overall motivation levels. We then computed person-specific Spearman rank correlations between the paired motivations across all available assessments. Fig. S12 shows the distribution of these person-specific correlations. Across participants, both motivational pairs exhibited negative correlations on average, indicating that moments of relatively lower motivation in one domain tended to co-occur with relatively higher motivation in the opposing domain. However, the strength of this coupling varied substantially between individuals. Thus, lower-than-usual motivation to exercise does not uniformly imply a higher-than-usual desire to rest, nor does lower-than-usual motivation to socialize necessarily imply a higher-than-usual wish to be alone.

Supplementary Figures

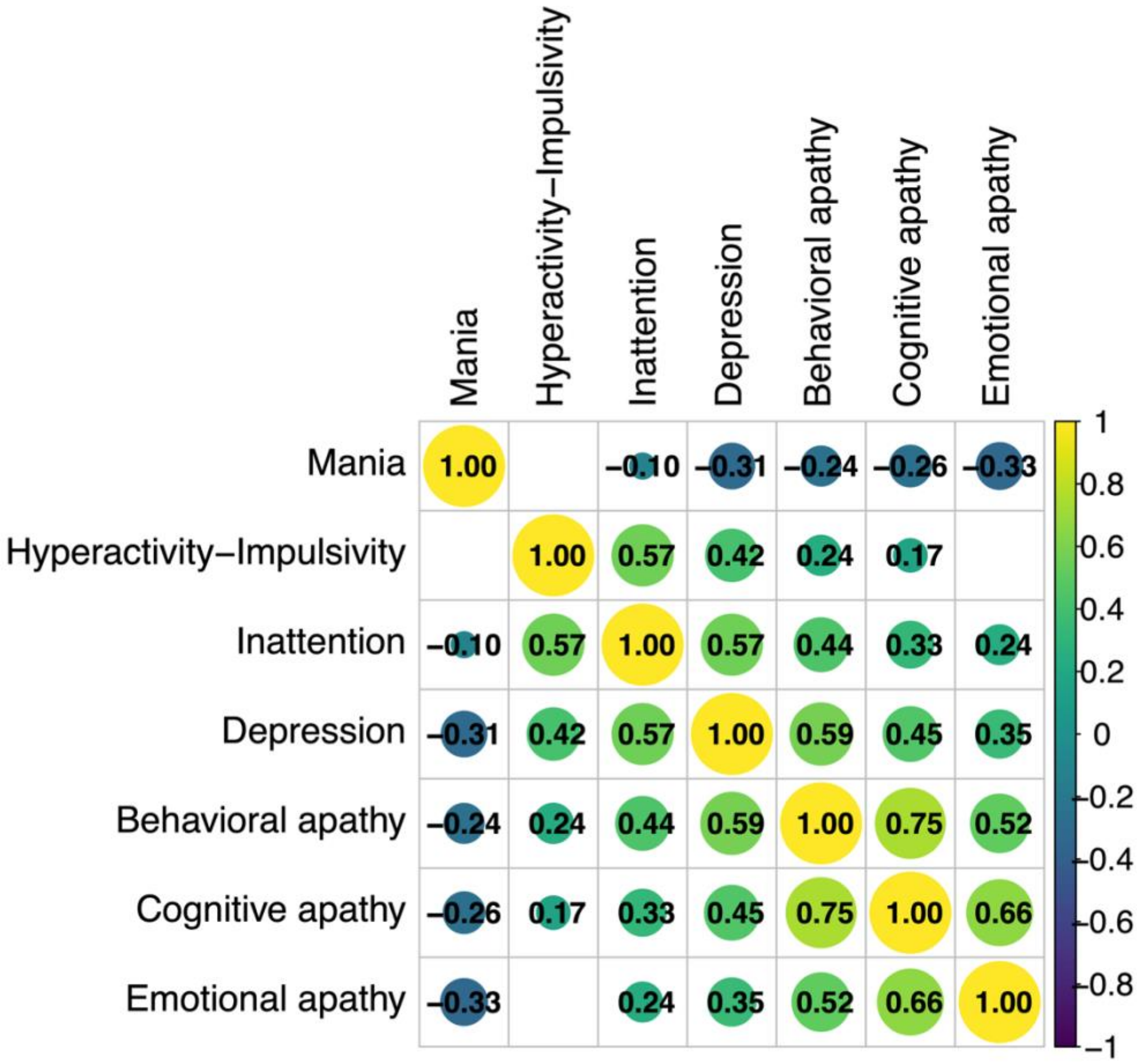

Fig. S1. Correlation matrix of the psychiatric scales

Spearman rank correlations between the psychiatric scales. For all scales, higher scores indicate greater symptom severity or prevalence. Correlation coefficients are shown within cells; blank cells indicate correlations with $p > 0.05$.

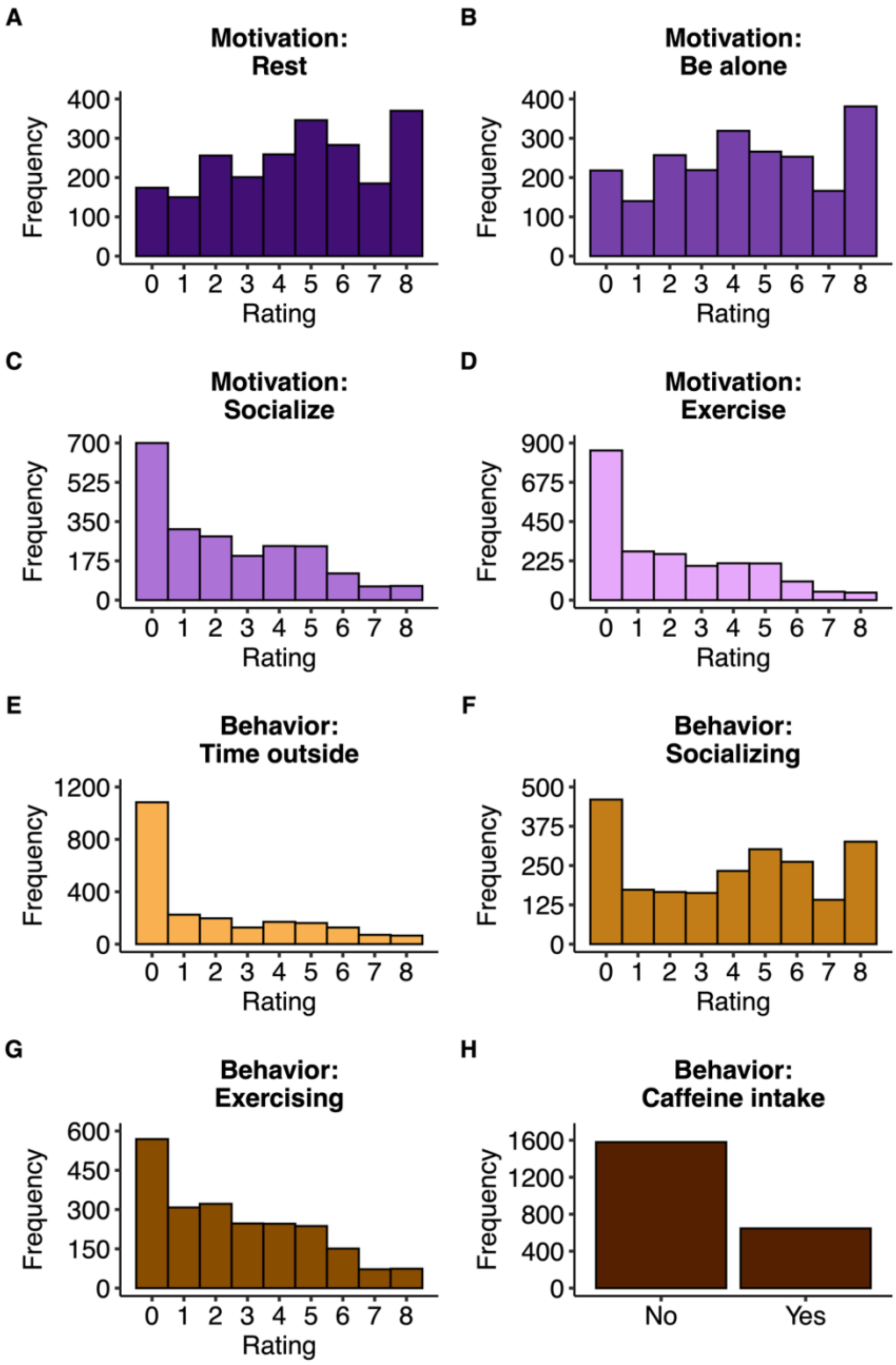

Fig. S2. Frequency distributions of motivation and behavior items

Items A–G are rated on a scale from 0 ("Not at all") to 8 ("Very much / All the time"). Item H represents a binary response: 0 = "No", 1 = "Yes".

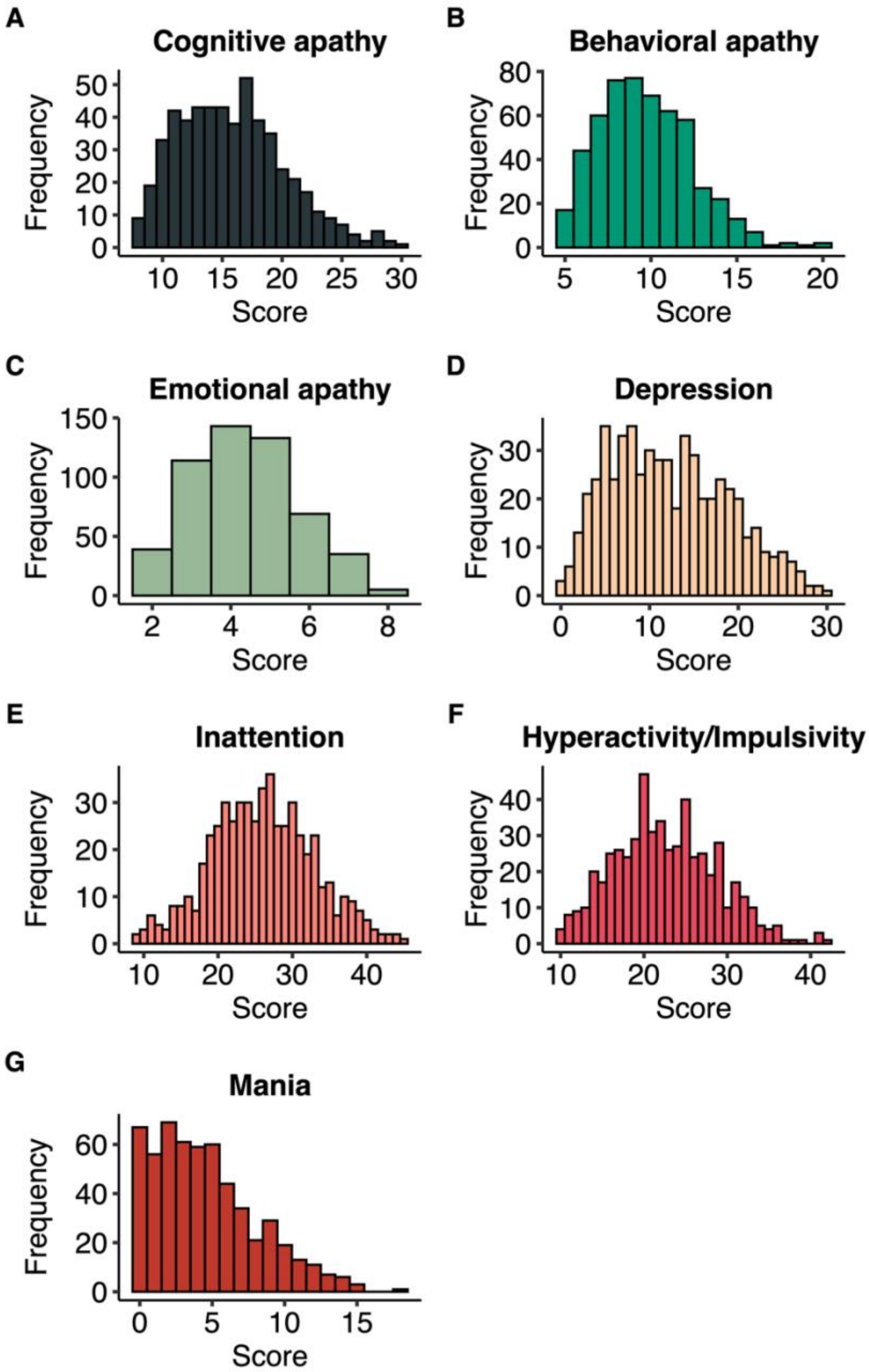

Fig. S3. Frequency plots of the psychiatric scale scores

Cognitive, behavioral, and emotional apathy (Apathy Evaluation Scale), depression (Center for Epidemiologic Studies Depression Scale Revised Short Form), inattention and hyperactivity/impulsivity (Adult ADHD Self-Report Scale), and mania (Altman Self-Rating Mania Scale).

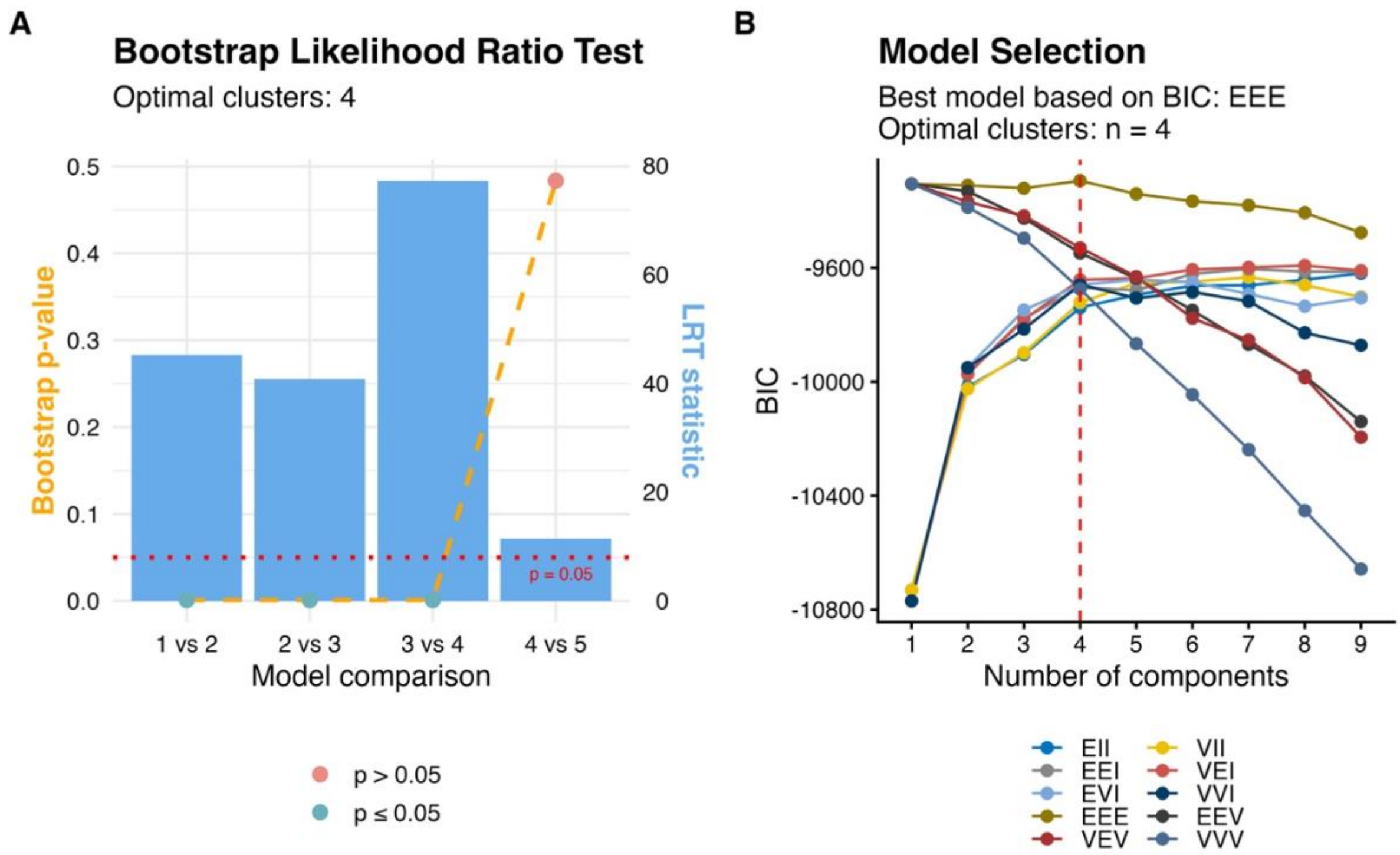

Fig. S4. Model selection indices for determining the optimal number of latent clusters

(A) Bootstrap Likelihood Ratio Test comparing models with G and G+1 clusters. Bars represent the likelihood ratio test (LRT) statistic (right y-axis), and the dashed yellow line shows the bootstrap p-value (left y-axis). The horizontal red dotted line marks the significance threshold ($p = 0.05$). The optimal number of clusters was determined as the highest G for which the model statistically significantly improved the fit. (B) Visual representation of the Bayesian Information Criterion (BIC) displaying the optimal number of clusters for different covariance structures and varying number of clusters. The red dashed line marks the optimal model. The BIC difference between the best model and the second-best model was 10.9, supporting the 4-cluster solution.

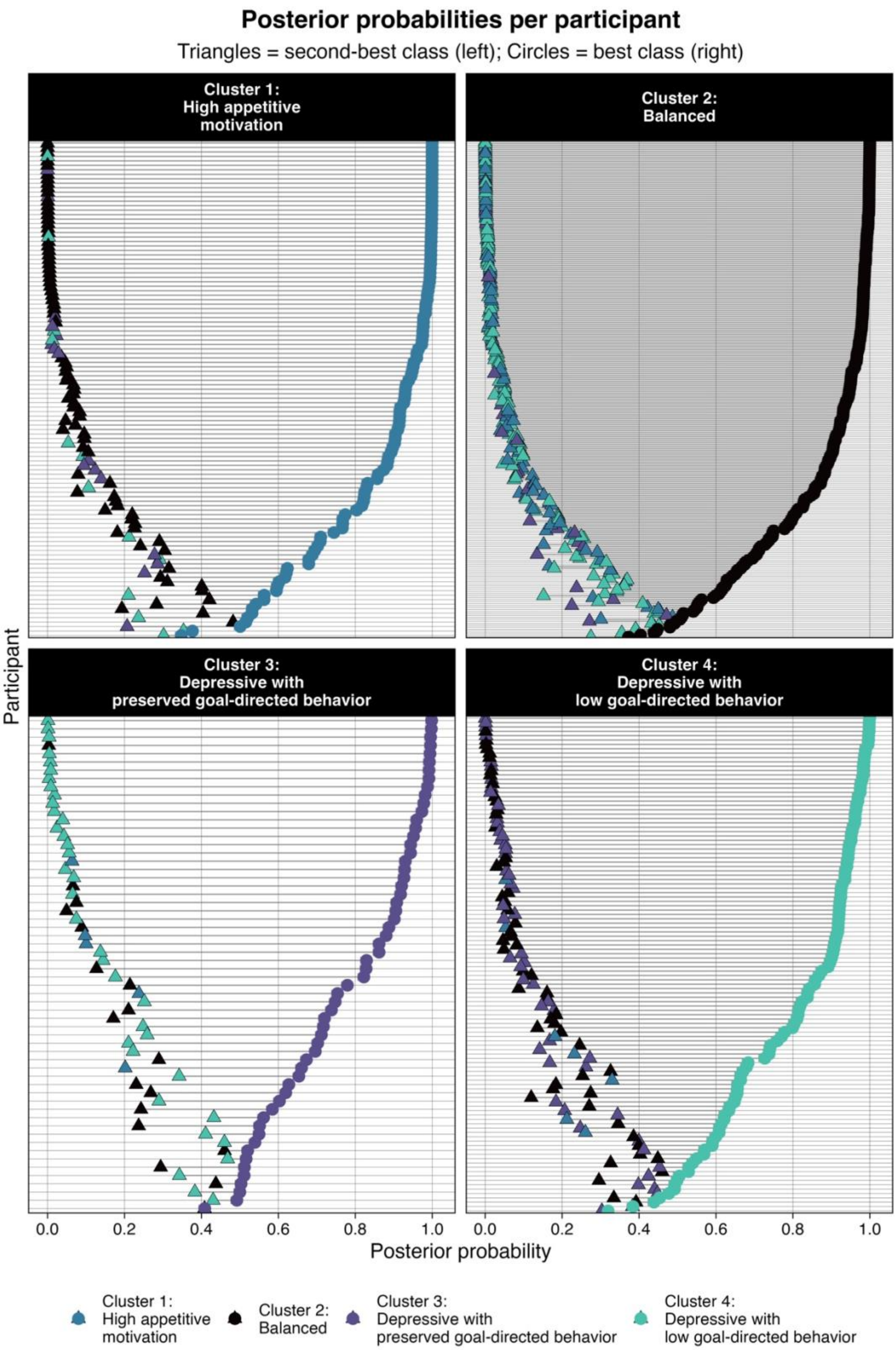

Fig. S5. Posterior classification probabilities for each participant across clusters

Posterior probabilities indicate the certainty with which each participant is assigned to a latent cluster. Within each panel, circles depict the posterior probability of belonging to the best-fitting (most likely) cluster, and triangles indicate the probability of belonging to the second-best cluster. Participants are sorted by their posterior probability within each cluster. High posterior probabilities (points near 1.0) reflect clear cluster separation and reliable classification. Lower probabilities suggest overlap or classification uncertainty between clusters.

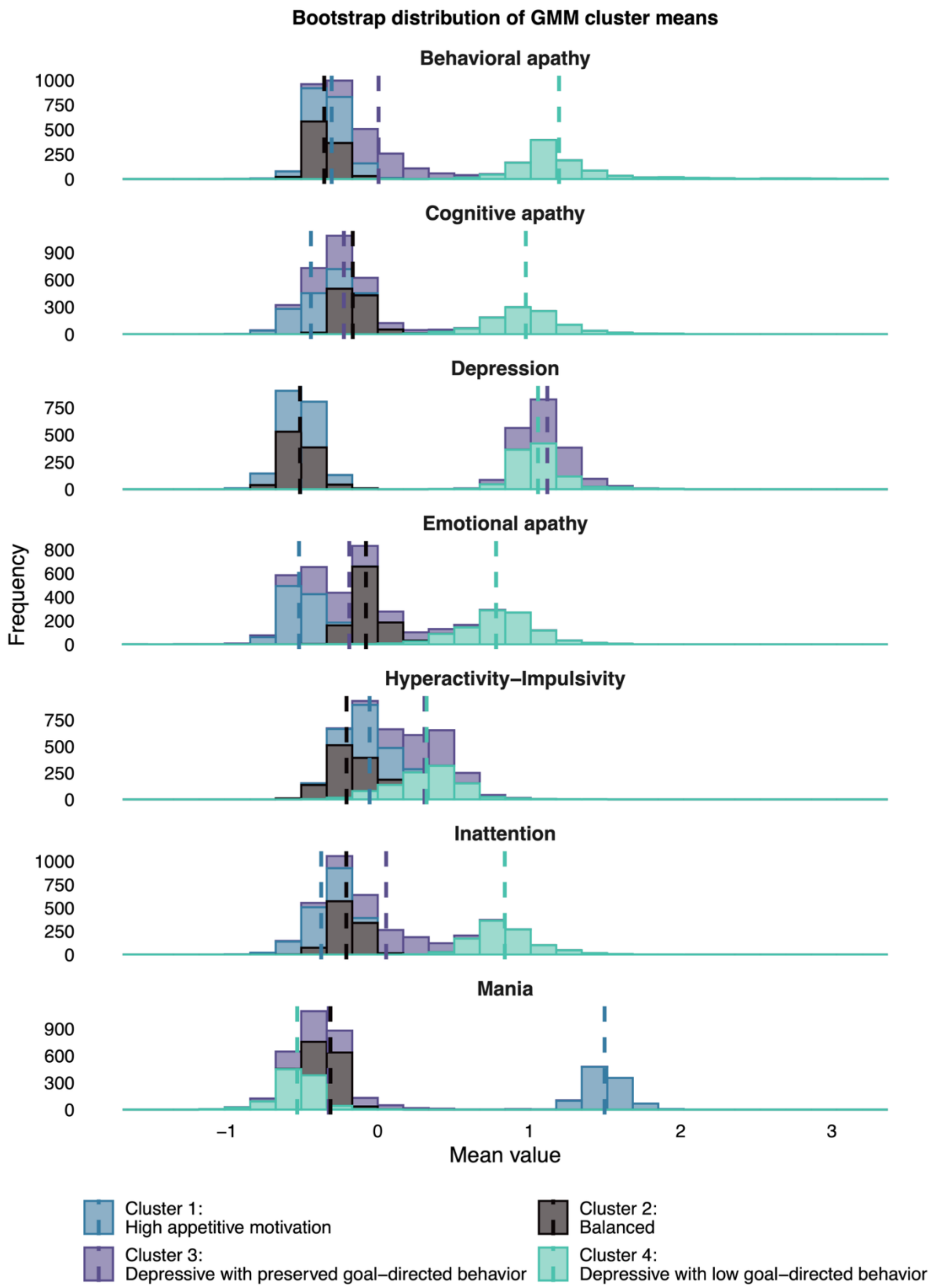

Fig. S6. Bootstrap distributions of GMM cluster means (parameter stability)

Each panel displays the distribution of bootstrapped mean estimates (n = 1000 nonparametric resamples) for one variable for each latent cluster. Vertical lines indicate the average estimate for each cluster and variable across bootstrap samples. The distributions reflect the stability and variability of the estimated cluster means, with tighter histograms indicating more robust parameter estimates across resamples. GMM = Gaussian Mixture Model.

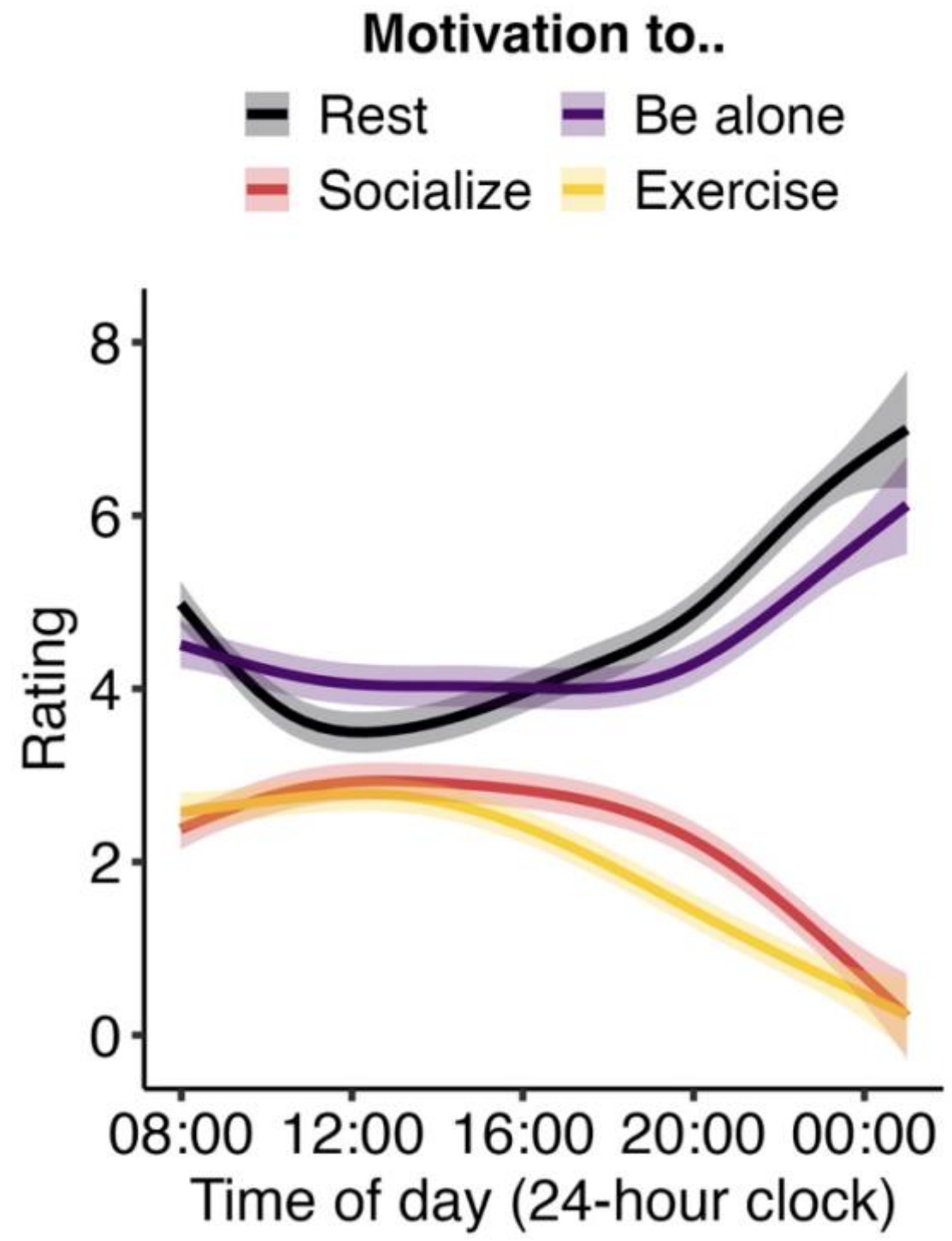

Fig. S7. Time-of-day patterns of motivation

The Y-axis represents the mean scores of item ratings (motivation to rest, be alone, exercise, and socialize across participants), where the scale ranges from 0 ("Not at all") to 8 ("Very much / All the time"). Error bands represent 95% pointwise confidence intervals. The presented values are predicted GAMM values.

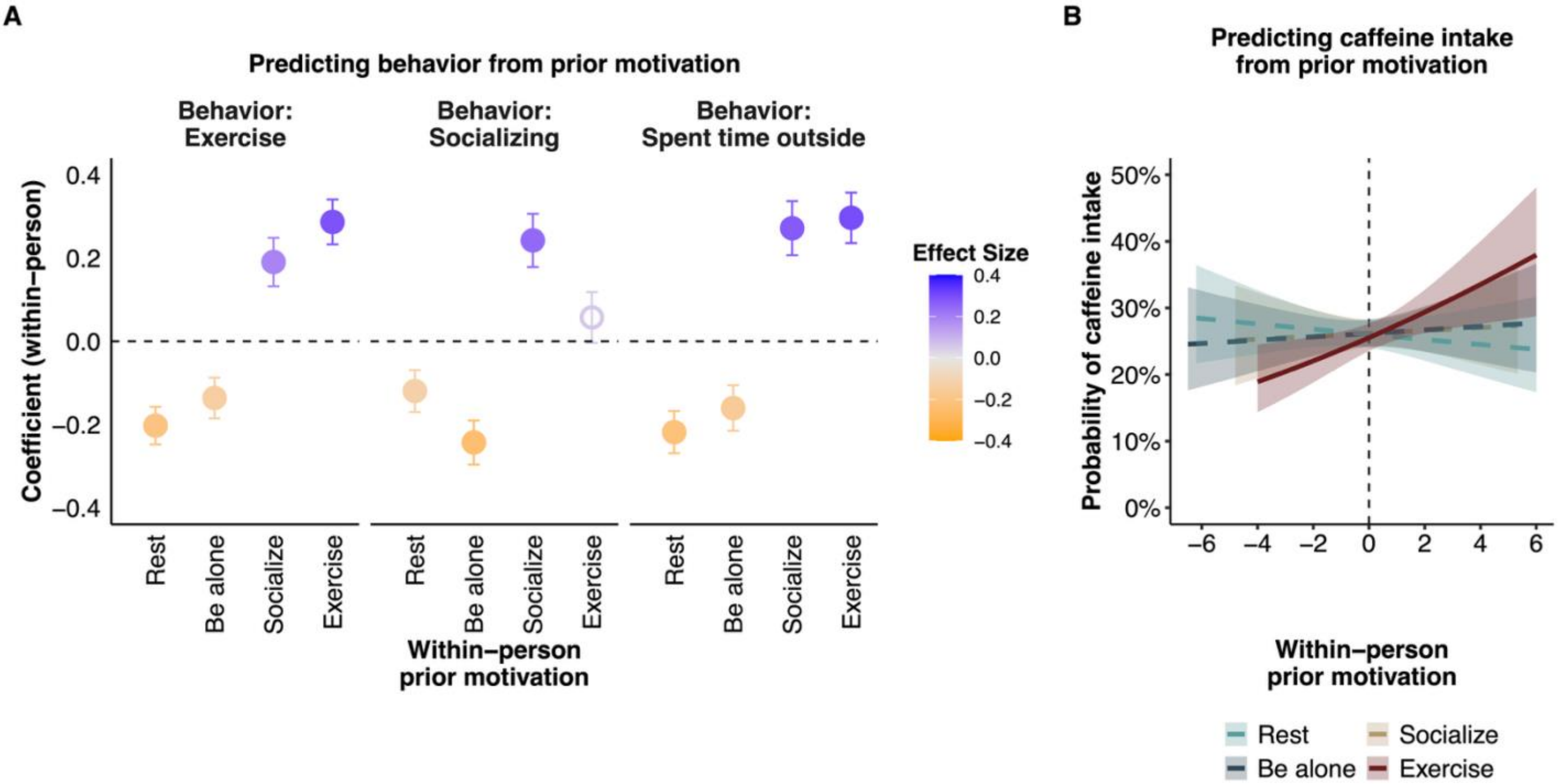

Fig. S8. Predicting behavior from prior motivation

(A) Coefficient plot showing the within-person effects of motivation on subsequent engagement in behaviors. A one-unit increase in an individual's motivation (relative to their personal mean) corresponds to the estimated change in behavior at the next time point, as indicated by the coefficient values. Solid circles denote $p < 0.05$, open circles indicate $p > 0.05$. (B) Predicted probability of caffeine intake based on prior motivation levels. The model estimates the probability of consuming caffeine as a function of previous motivation. A dashed line indicates $p > 0.05$, solid line $p < 0.05$. Error bars and bands represent 95% confidence intervals.

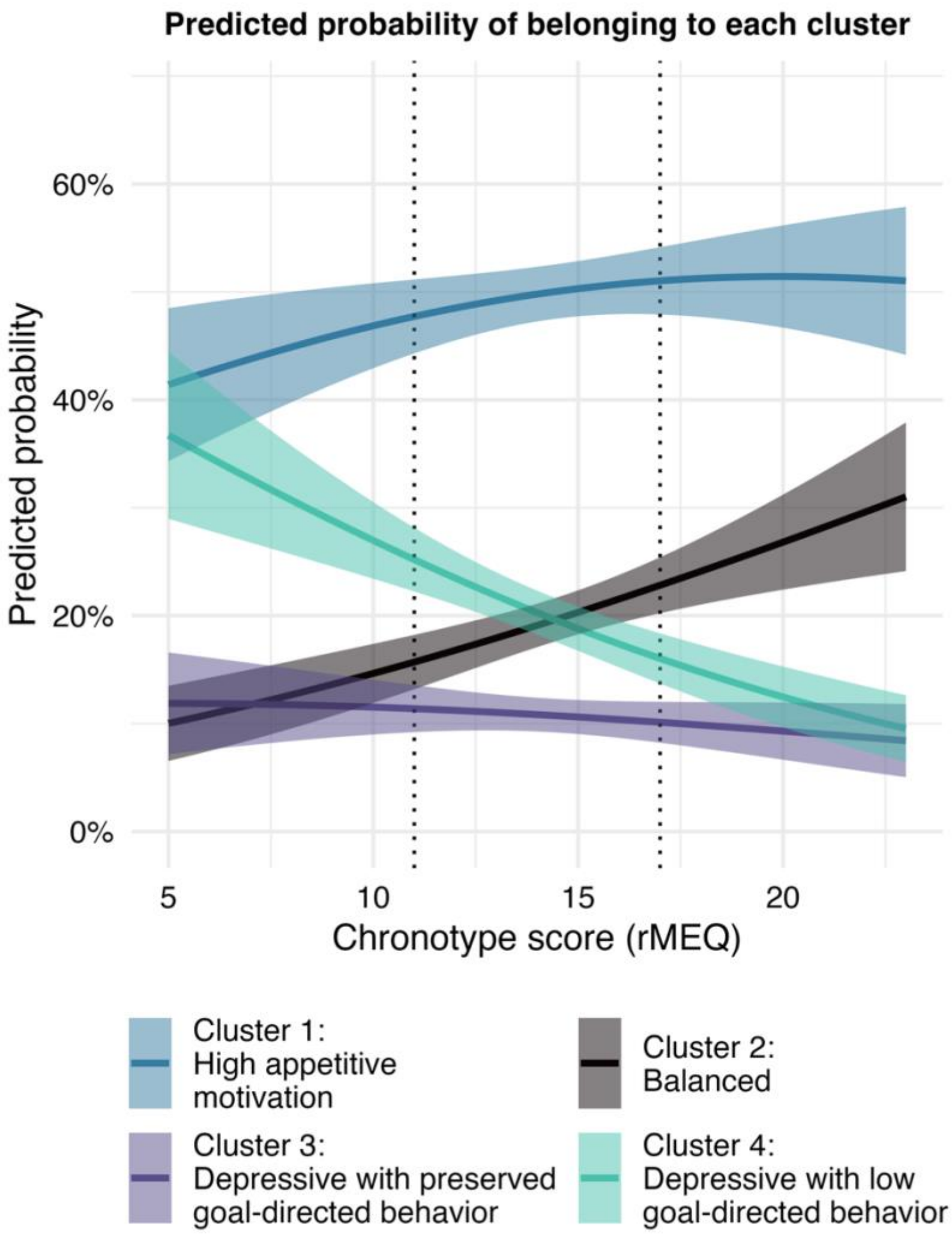

Fig. S9. Association between cluster membership and chronotype

Predicted probabilities of belonging to each cluster as a function of chronotype, measured by the reduced Morningness Eveningness Questionnaire [rMEQ, estimated using multinomial logistic regression. Error bands represent 95% confidence intervals. Higher rMEQ scores reflect stronger morningness. Membership in the *high appetitive motivation* and *balanced* clusters increased with stronger morningness, whereas membership of the *Depressive with low goal-directed behavior* cluster was less likely as chronotype is later (lower rMEQ scores). Vertical dotted lines mark the standard cut-offs for evening and morning chronotypes

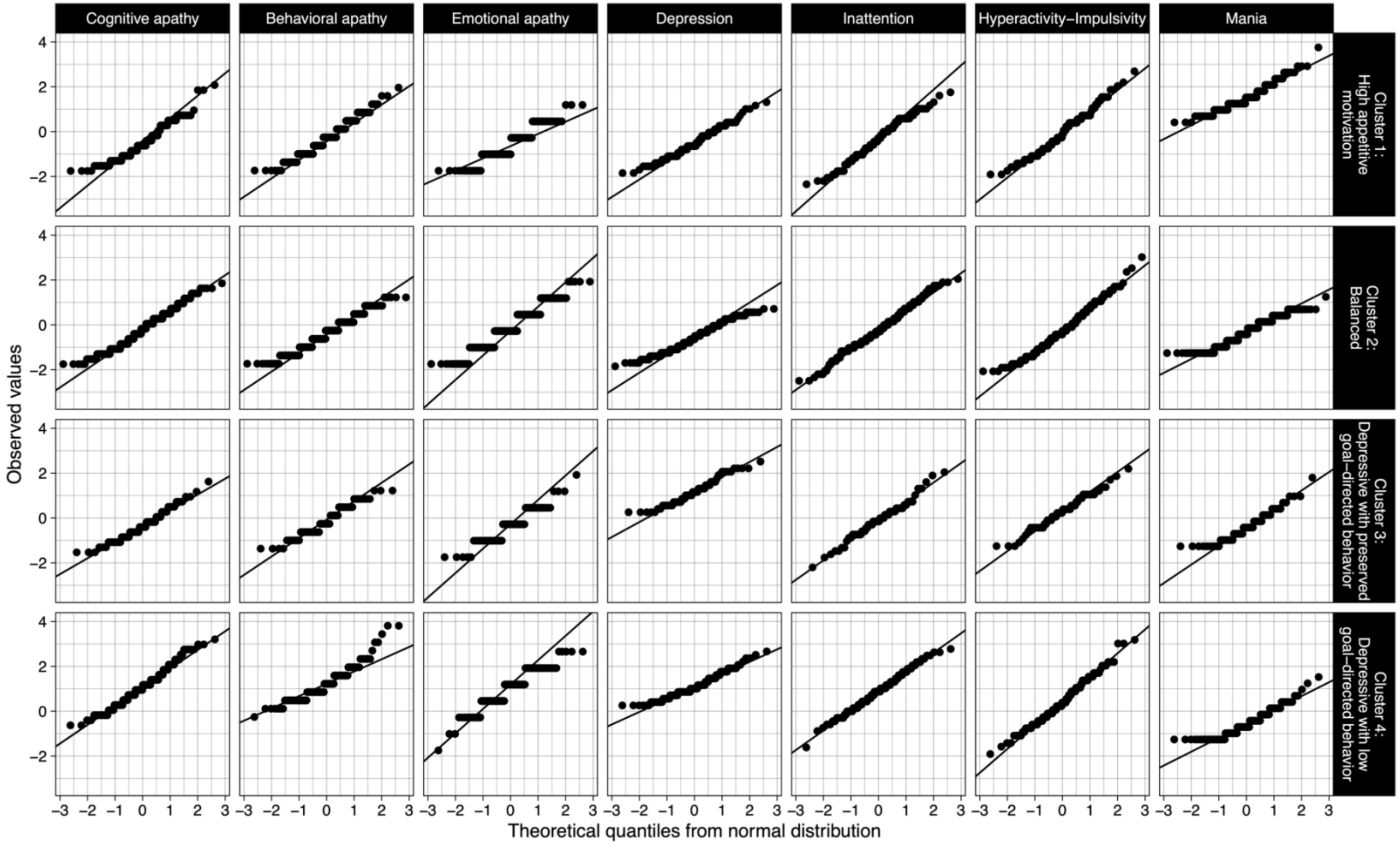

Fig. S10. Normal Q–Q plots of trait variables by latent cluster

Panels show quantile–quantile (Q–Q) plots comparing observed values (y-axis) with theoretical quantiles from a normal distribution (x-axis) for each variable used in the clustering analysis: cognitive apathy, behavioral apathy, emotional apathy, depression, inattention, hyperactivity/impulsivity, and mania. Rows correspond to the four identified latent clusters: Cluster 1 (high appetitive motivation), Cluster 2 (balanced), Cluster 3 (depressive with preserved goal-directed behavior), and Cluster 4 (depressive with low goal-directed behavior). Deviations from the diagonal reference line reflect departures from normality within clusters and variables.

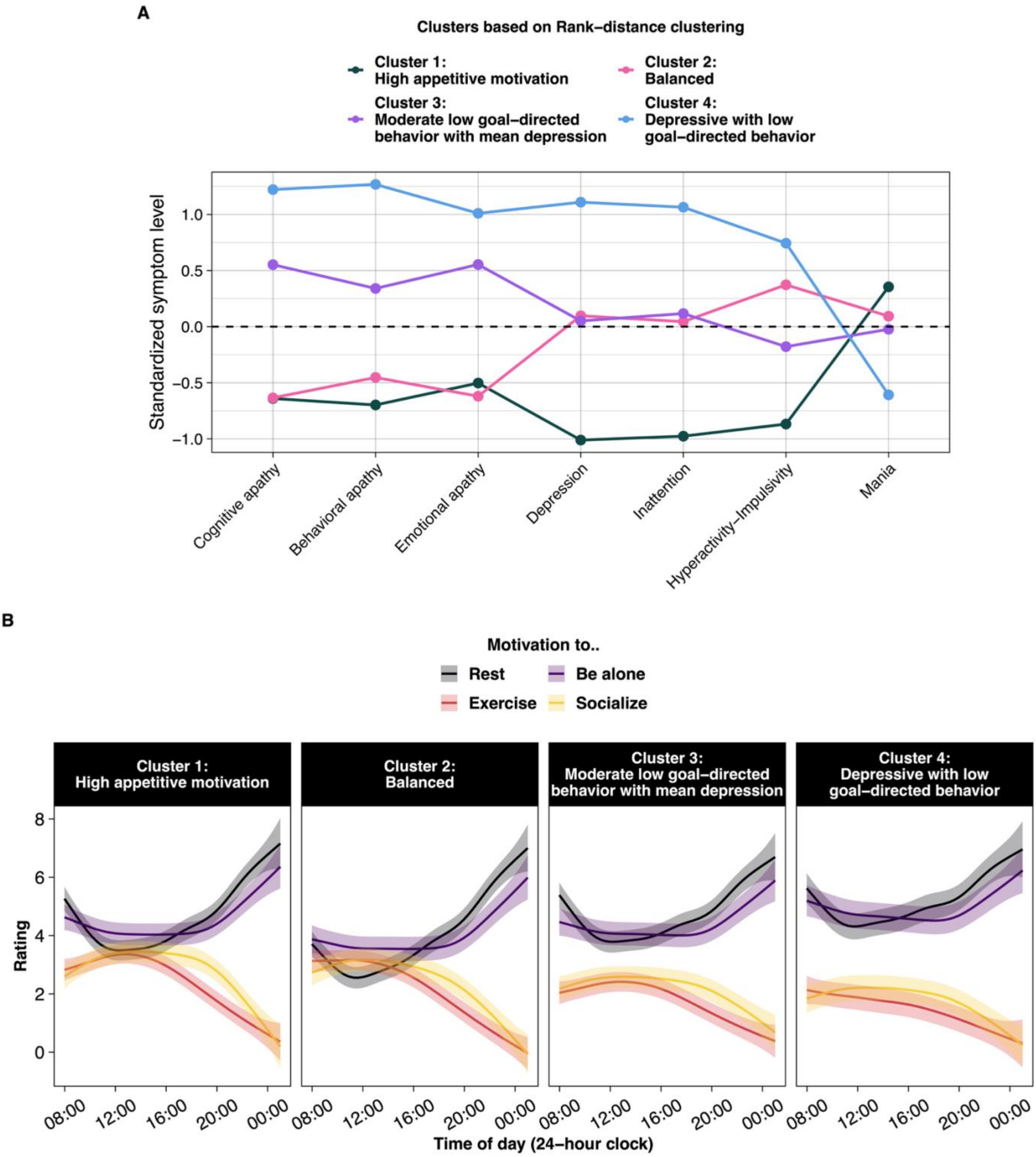

Fig. S11. Rank-distance clustering and corresponding diurnal motivation patterns
(A) Standardized levels of cognitive apathy, behavioral apathy, emotional apathy, depression, inattention, hyperactivity/impulsivity, and mania for the four clusters as obtained using rank-distance hierarchical clustering. Values were standardized relative to the full sample. (B) Cluster-specific diurnal trajectories of momentary motivation to rest, be alone, exercise, and socialize across the day. Solid lines represent estimated smooth functions from generalized additive mixed models, and shaded areas represent 95% confidence intervals. Time of day is shown on a 24-hour clock.

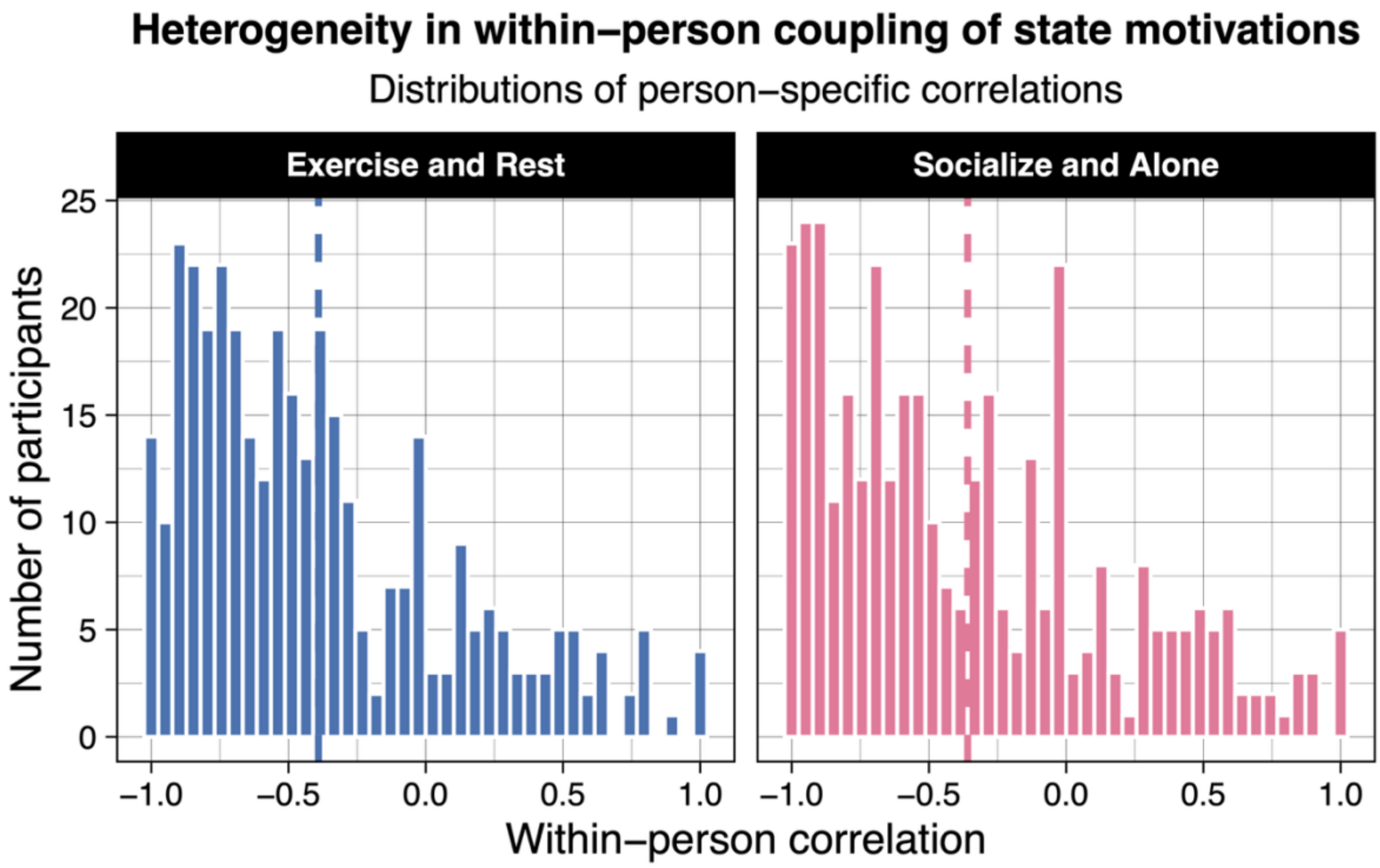

Fig. S12. Distribution of within-person correlations of state motivations
Distributions of person-specific within-person Spearman correlations between motivation to exercise and motivation to rest (left panel), and between motivation to socialize and motivation to be alone (right panel). Each bar represents the number of participants exhibiting a given correlation value. Correlations were computed separately for each individual using person-mean centered motivation ratings, thereby indexing within-person coupling. Dotted vertical lines indicate the mean correlation for each motivational pair.

Supplementary Tables

Table S1. Age and gender distribution in the full sample (N = 538)

The full sample used in the multivariate GMM (cluster analysis).

**TOTAL SAMPLE N = 538**	**Cluster 1 High appetitive motivation**	**Cluster 2 Balanced**	**Cluster 3 Depressive, preserved goal-directed behavior**	**Cluster 4 Depressive, low goal-directed behavior**
N	111	253	60	114
Age, mean (SD)	28.8 (7.1)	34.4 (10.0)	31.2 (8.1)	31.8 (10.0)
Gender, count (%)				
Women	87 (78.4%)	192 (75.9%)	54 (90.0%)	91 (79.8%)
Men	23 (20.7%)	60 (23.7%)	6 (10.0%)	23 (20.2%)
Non-binary	1 (0.9%)	1 (0.4%)	0	0

Table S2. Age and gender distribution in the EMA subsample (N = 410)

**EMA SAMPLE N = 410**	**Cluster 1 High appetitive motivation**	**Cluster 2 Balanced**	**Cluster 3 Depressive, preserved goal-directed behavior**	**Cluster 4 Depressive, low goal-directed behavior**
N	81	202	44	83
Age, mean (SD)	28.7 (7.1)	33.7 (9.5)	31.9 (8.0)	32.3 (9.2)
Gender, count (%)				
Women	59 (72.8%)	152 (75.2%)	40 (90.9%)	69 (83.1%)
Men	21 (25.9%)	49 (24.3%)	4 (9.1%)	14 (16.9%)
Non-binary	1 (1.2%)	1 (0.5%)	0	0

Table S3. Generalized additive mixed model (GAMM) results predicting Wanting to rest

Want to rest				
Parametric coefficients	Estimate	SE	T value	p-value
Intercept	4.37	0.18	24.38	<2e-16***
Cluster 2	-0.08	0.21	-0.39	0.697
Cluster 3	0.37	0.30	1.22	0.224
Cluster 4	0.57	0.25	2.27	0.024*
Smooth terms	EDF	RefDF	F value	p-value
ID	301.31	406.00	2.97	<2e-16***
Time of day	4.83	5.90	37.92	<2e-16***
Time of day x Cluster 1	1.97	2.44	1.33	0.322
Time of day x Cluster 2	1.00	1.00	0.46	0.497
Time of day x Cluster 3	1.00	1.00	1.48	0.223
Time of day x Cluster 4	1.68	2.08	0.94	0.402

R-sq.(adj) = 0.44 Deviance explained = 52.2%
REML = 4849 Scale est. = 3.48 n = 2224
Cluster definitions: Cluster 1 = High appetitive motivation, Cluster 2 = Balanced, Cluster 3 = Depressive-preserved motivation, Cluster 4 = Depressive-low motivation.
The null hypothesis for the smooth function is that the term is constant across time of day (i.e., the smooth is a flat line). The effective degrees of freedom (EDF) reflect the shape of the smooth: EDF ≈ 1 indicates an approximately linear effect and an EDF > 1 suggests a non-linear pattern.

Table S4. GAMM results predicting Wanting to be alone

Want to be alone				
Parametric coefficients	Estimate	SE	T value	p-value
Intercept	4.32	0.21	20.85	<2e-16***
Cluster 2	-0.26	0.25	-1.07	0.285
Cluster 3	0.30	0.35	0.87	0.386
Cluster 4	0.70	0.29	2.41	0.016*
Smooth terms	EDF	RefDF	F value	p-value
ID	327.92	406.00	4.44	<2e-16***
Time of day	2.64	3.46	5.07	0.001***
Time of day x Cluster 1	2.19	2.71	1.45	0.248
Time of day x Cluster 2	3.35	4.07	2.94	0.018
Time of day x Cluster 3	1.00	1.00	1.53	0.217
Time of day x Cluster 4	1.00	1.00	0.51	0.475

R-sq.(adj) = 0.48 Deviance explained = 55.8%
REML = 4885 Scale est. = 3.44 n = 2219
Cluster definitions: Cluster 1 = High appetitive motivation, Cluster 2 = Balanced, Cluster 3 = Depressive-preserved motivation, Cluster 4 = Depressive-low motivation.
The null hypothesis for the smooth function is that the term is constant across time of day (i.e., the smooth is a flat line). The effective degrees of freedom (EDF) reflect the shape of the smooth: EDF ≈ 1 indicates an approximately linear effect and an EDF > 1 suggests a non-linear pattern.

Table S5. GAMM results predicting Wanting to socialize

Want to socialize				
Parametric coefficients	Estimate	SE	T value	p-value

Intercept	3.06	0.19	15.88	<2e-16***
Cluster 2	-0.59	0.23	-2.59	0.010**
Cluster 3	-0.74	0.33	-2.26	0.024*
Cluster 4	-1.38	0.27	-5.10	0.000***
Smooth terms	EDF	RefDF	F value	p-value
ID	344.98	406.00	5.84	<2e-16***
Time of day	3.78	4.78	23.52	<2e-16***
Time of day x Cluster 1	1.00	1.00	3.96	0.047
Time of day x Cluster 2	1.48	1.79	2.76	0.118
Time of day x Cluster 3	1.00	1.00	3.78	0.052
Time of day x Cluster 4	2.42	2.98	2.31	0.073

R-sq.(adj) = 0.56 Deviance explained = 63.3%
REML = 4506 Scale est. = 2.33 n = 2221
Cluster definitions: Cluster 1 = High appetitive motivation, Cluster 2 = Balanced, Cluster 3 = Depressive-preserved motivation, Cluster 4 = Depressive-low motivation.
The null hypothesis for the smooth function is that the term is constant across time of day (i.e., the smooth is a flat line). The effective degrees of freedom (EDF) reflect the shape of the smooth: EDF ≈ 1 indicates an approximately linear effect and an EDF > 1 suggests a non-linear pattern.

Table S6. GAMM results predicting Wanting to exercise

Want to exercise				
Parametric coefficients	Estimate	SE	T value	p-value
Intercept	2.73	0.18	15.53	<2e-16***
Cluster 2	-0.75	0.21	-3.60	0.000***
Cluster 3	-0.38	0.30	-1.29	0.197
Cluster 4	-1.10	0.25	-4.46	0.000***
Smooth terms	EDF	RefDF	F value	p-value
ID	333.09	406.00	4.76	<2e-16***
Time of day	2.46	3.25	7.91	0.000***
Time of day x Cluster 1	1.18	1.32	5.53	0.020*
Time of day x Cluster 2	2.36	2.90	2.71	0.055
Time of day x Cluster 3	3.07	3.75	3.69	0.009**
Time of day x Cluster 4	1.00	1.00	1.36	0.243

R-sq.(adj) = 0.54 Deviance explained = 61.2%
REML = 4447 Scale est. = 2.30 n = 2215
Cluster definitions: Cluster 1 = High appetitive motivation, Cluster 2 = Balanced, Cluster 3 = Depressive-preserved motivation, Cluster 4 = Depressive-low motivation.
The null hypothesis for the smooth function is that the term is constant across time of day (i.e., the smooth is a flat line). The effective degrees of freedom (EDF) reflect the shape of the smooth: EDF ≈ 1 indicates an approximately linear effect and an EDF > 1 suggests a non-linear pattern.

Table S7. GAMM results predicting Time outside

Time outside				
Parametric coefficients	Estimate	SE	T value	p-value
Intercept	2.50	0.16	15.98	<2e-16***
Cluster 2	-0.60	0.18	-3.25	0.001**
Cluster 3	-0.68	0.26	-2.57	0.010*
Cluster 4	-0.92	0.22	-4.21	0.000***
Smooth terms	EDF	RefDF	F value	p-value
ID	236.46	406.00	1.43	<2e-16***
Time of day	4.09	5.12	29.91	<2e-16***
Time of day x Cluster 1	1.00	1.00	2.13	0.145
Time of day x Cluster 2	1.00	1.00	1.75	0.186
Time of day x Cluster 3	1.00	1.00	1.88	0.170
Time of day x Cluster 4	1.00	1.00	1.80	0.180

R-sq.(adj) = 0.26 Deviance explained = 33.8%
REML = 4995 Scale est. = 4.33 n = 2226
Cluster definitions: Cluster 1 = High appetitive motivation, Cluster 2 = Balanced, Cluster 3 = Depressive-preserved motivation, Cluster 4 = Depressive-low motivation.
The null hypothesis for the smooth function is that the term is constant across time of day (i.e., the smooth is a flat line). The effective degrees of freedom (EDF) reflect the shape of the smooth: EDF ≈ 1 indicates an approximately linear effect and an EDF > 1 suggests a non-linear pattern.

Table S8. GAMM results predicting Socializing behavior

Socialize				
Parametric coefficients	Estimate	SE	T value	p-value
Intercept	4.07	0.22	18.61	<2e-16***
Cluster 2	0.00	0.26	0.01	0.995
Cluster 3	-0.75	0.37	-2.05	0.041*
Cluster 4	-0.62	0.31	-2.02	0.043*
Smooth terms	EDF	RefDF	F value	p-value
ID	312.96	406.00	3.58	<2e-16***
Time of day	4.32	5.37	3.93	0.003**
Time of day x Cluster 1	1.82	2.26	2.14	0.116
Time of day x Cluster 2	1.00	1.00	2.55	0.110
Time of day x Cluster 3	1.00	1.00	2.34	0.127
Time of day x Cluster 4	1.00	1.00	3.20	0.074

R-sq.(adj) = 0.42 Deviance explained = 50.3%
REML = 5185 Scale est. = 4.59 n = 2226
Cluster definitions: Cluster 1 = High appetitive motivation, Cluster 2 = Balanced, Cluster 3 = Depressive-preserved motivation, Cluster 4 = Depressive-low motivation.
The null hypothesis for the smooth function is that the term is constant across time of day (i.e., the smooth is a flat line). The effective degrees of freedom (EDF) reflect the shape of the smooth: EDF ≈ 1 indicates an approximately linear effect and an EDF > 1 suggests a non-linear pattern.

Table S9. GAMM results predicting Exercising

Exercising				
Parametric coefficients	Estimate	SE	T value	p-value
Intercept	3.09	0.17	18.44	<2e-16***
Cluster 2	-0.44	0.20	-2.24	0.025*
Cluster 3	-0.54	0.28	-1.92	0.055
Cluster 4	-0.78	0.23	-3.32	0.001***
Smooth terms	EDF	RefDF	F value	p-value
ID	285.97	406.00	2.50	<2e-16***
Time of day	3.60	4.57	30.49	<2e-16***
Time of day x Cluster 1	1.00	1.00	0.31	0.575
Time of day x Cluster 2	1.00	1.00	0.06	0.811
Time of day x Cluster 3	1.00	1.00	0.13	0.717
Time of day x Cluster 4	1.73	2.14	0.70	0.498

R-sq.(adj) = 0.35 Deviance explained = 43.6%
REML = 4826 Scale est. = 3.49 n = 2226
Cluster definitions: Cluster 1 = High appetitive motivation, Cluster 2 = Balanced, Cluster 3 = Depressive-preserved motivation, Cluster 4 = Depressive-low motivation.
The null hypothesis for the smooth function is that the term is constant across time of day (i.e., the smooth is a flat line). The effective degrees of freedom (EDF) reflect the shape of the smooth: EDF ≈ 1 indicates an approximately linear effect and an EDF > 1 suggests a non-linear pattern.

Table S10. GAMM results predicting Caffeine intake

Caffeine				
Parametric coefficients	OR	95% CI		p-value
Intercept	0.22	0.15-0.32		<2e-16***
Cluster 2	1.43	0.92-2.23		0.114
Cluster 3	1.97	1.06-3.65		0.032*
Cluster 4	2.22	1.33-3.69		0.002**
Smooth terms	EDF	RefDF	Chi.sq	p-value
ID	222.43	406.00	485.59	<2e-16***
Time of day	0.55	0.90	0.01	0.931
Time of day x Cluster 1	3.08	3.77	10.05	0.035*
Time of day x Cluster 2	1.00	1.00	19.24	0.000***
Time of day x Cluster 3	1.00	1.00	18.43	0.000***
Time of day x Cluster 4	2.16	2.66	7.46	0.058

R-sq.(adj) = 0.32 Deviance explained = 33.5%
REML = 1191 Scale est. = 1 n = 2226
Cluster definitions: Cluster 1 = High appetitive motivation, Cluster 2 = Balanced, Cluster 3 = Depressive-preserved motivation, Cluster 4 = Depressive-low motivation.
The null hypothesis for the smooth function is that the term is constant across time of day (i.e., the smooth is a flat line). The effective degrees of freedom (EDF) reflect the shape of the smooth: EDF ≈ 1 indicates an approximately linear effect and an EDF > 1 suggests a non-linear pattern.

Table S11. GAMM results predicting Socializing from motivation

Social motivation → Socializing				
Parametric coefficients	Estimate	SE	T value	p-value
Intercept	0.26	0.13	2.09	0.037*
Cluster 2	-0.03	0.14	-0.19	0.846
Cluster 3	0.20	0.21	0.99	0.321
Cluster 4	-0.03	0.16	-0.21	0.835
Smooth terms	EDF	RefDF	F value	p-value
ID	0.00	405.00	0.00	1.000
Social motivation	1.80	5.00	4.05	3.9e-06***
Time of day	2.39	5.00	4.49	<2e-16***
Time of day x Social motivation	6.76	16.00	1.82	9.3e-06***
Time of day x Cluster 1	0.68	4.00	0.54	0.052
Time of day x Cluster 2	0.87	4.00	0.46	0.087
Time of day x Cluster 3	1.42	4.00	0.88	0.072
Time of day x Cluster 4	0.00	4.00	0.00	0.838
Social motivation x Cluster 1	1.35	4.00	1.54	0.010**
Social motivation x Cluster 2	0.00	4.00	0.00	0.693
Social motivation x Cluster 3	1.58	4.00	2.30	0.002**
Social motivation x Cluster 4	0.00	4.00	0.00	0.885
Time of day x Social motivation x Cluster 1	0.14	8.00	0.02	0.293
Time of day x Social motivation x Cluster 2	0.00	9.00	0.00	0.439
Time of day x Social motivation x Cluster 3	0.76	8.00	0.17	0.152
Time of day x Social motivation x Cluster 4	0.00	9.00	0.00	0.446

R-sq.(adj) = 0.09 Deviance explained = 9.9%
fREML = 3700 Scale est. = 3.38 n = 1811
Cluster definitions: Cluster 1 = High appetitive motivation, Cluster 2 = Balanced, Cluster 3 = Depressive-preserved motivation, Cluster 4 = Depressive-low motivation.

EDF values of 0 indicate terms that were fully penalized toward zero under shrinkage.

Table S12. GAMM results predicting Exercising from Exercise motivation

Exercise motivation → Exercising				
Parametric coefficients	Estimate	SE	T value	p-value
Intercept	0.10	0.09	1.18	0.237
Cluster 2	0.10	0.11	0.91	0.362
Cluster 3	0.15	0.15	1.00	0.317
Cluster 4	0.04	0.12	0.3	0.761
Smooth terms	EDF	RefDF	F value	p-value
ID	0.00	405.00	0.00	1.000
Exercise motivation	0.97	5.00	6.94	<2e-16***
Time of day	3.01	5.00	16.62	<2e-16***
Time of day x Exercise motivation	1.02	16.00	0.12	0.089
Time of day x Cluster 1	0.00	4.00	0.00	0.558
Time of day x Cluster 2	0.68	4.00	0.53	0.067
Time of day x Cluster 3	0.00	4.00	0.00	0.352
Time of day x Cluster 4	0.00	4.00	0.00	0.639
Exercise motivation x Cluster 1	0.00	4.00	0.00	0.841
Exercise motivation x Cluster 2	0.66	4.00	0.28	0.176
Exercise motivation x Cluster 3	0.00	4.00	0.00	0.661
Exercise motivation x Cluster 4	0.00	4.00	0.00	0.352

Time of day x Exercise motivation x Cluster 1	0.00	9.00	0.00	0.564
Time of day x Exercise motivation x Cluster 2	1.00	9.00	0.37	0.031*
Time of day x Exercise motivation x Cluster 3	0.75	9.00	0.11	0.255
Time of day x Exercise motivation x Cluster 4	1.74	9.00	0.63	0.027*

R-sq.(adj) = 0.11 Deviance explained = 11.5%
fREML = 3492 Scale est. = 2.72 n = 1809
Cluster definitions: Cluster 1 = High appetitive motivation, Cluster 2 = Balanced, Cluster 3 = Depressive-preserved motivation, Cluster 4 = Depressive-low motivation.
EDF values of 0 indicate terms that were fully penalized toward zero under shrinkage.

Table S13. Percentage of missing EMA observations at each timepoint by cluster and data collection ID

Data collection ID 0 included five timepoints (distributed between ~8:00 and 00:00). Data collection ID 1 included six timepoints (distributed between ~8:00 and 23:00); therefore, Timepoint 6 is not applicable for data collection ID 0.

Data collection ID	Cluster	Timepoint 1	Timepoint 2	Timepoint 3	Timepoint 4	Timepoint 5	Timepoint 6
0	1	0.0%	0.0%	0.0%	0.0%	10.0%	Not applicable
0	2	0.0%	5.0%	10.0%	0.0%	10.0%	Not applicable
0	3	0.0%	0.0%	0.0%	14.3%	14.3%	Not applicable
0	4	0.0%	10.0%	10.0%	0.0%	0.0%	Not applicable
1	1	5.6%	11.3%	7.0%	9.9%	14.1%	7.0%
1	2	7.7%	3.8%	8.2%	7.7%	8.2%	14.3%
1	3	5.4%	2.7%	8.1%	8.1%	10.8%	16.2%
1	4	5.5%	4.1%	5.5%	9.6%	6.8%	5.5%

Table S14. Comparison of smooth terms between primary GAMMs and AR(1) sensitivity models for motivation EMA items

Effective Degrees of Freedom (EDFs), F test statistics, and p-values are shown.

Item	Term	EDF main model	EDF AR1 model	F main model	F AR1 model	*p* main model	*p* AR1 model
Rest	s(id)	301.31	285.84	2.97	2.43	0.000	0.000
Rest	s(Time of day)	4.83	4.95	37.92	37.45	0.000	0.000
Rest	s(Time of day):cluster1	1.97	1.99	1.33	1.03	0.322	0.323
Rest	s(Time of day):cluster2	1.00	1.00	0.46	0.45	0.497	0.505
Rest	s(Time of day):cluster3	1.00	1.00	1.48	1.48	0.223	0.224
Rest	s(Time of day):cluster4	1.68	1.59	0.94	0.90	0.402	0.457
Self	s(id)	327.92	329.90	4.44	4.59	0.000	0.000
Self	s(Time of day)	2.64	2.62	5.07	5.08	0.001	0.001
Self	s(Time of day):cluster1	2.19	2.19	1.45	1.45	0.248	0.244
Self	s(Time of day):cluster2	3.35	3.34	2.94	2.95	0.018	0.018
Self	s(Time of day):cluster3	1.00	1.00	1.53	1.54	0.217	0.215
Self	s(Time of day):cluster4	1.00	1.00	0.51	0.51	0.475	0.475
Socialize	s(id)	344.98	341.47	5.84	5.41	0.000	0.000
Socialize	s(Time of day)	3.78	3.82	23.52	23.45	0.000	0.000
Socialize	s(Time of day):cluster1	1.00	1.00	3.96	3.92	0.047	0.048
Socialize	s(Time of day):cluster2	1.48	1.47	2.76	2.75	0.118	0.120
Socialize	s(Time of day):cluster3	1.00	1.00	3.78	3.73	0.052	0.053
Socialize	s(Time of day):cluster4	2.42	2.46	2.31	2.31	0.073	0.074
Exercise	s(id)	333.09	328.02	4.76	4.36	0.000	0.000
Exercise	s(Time of day)	2.46	2.52	7.91	8.45	0.000	0.000
Exercise	s(Time of day):cluster1	1.18	1.07	5.53	5.92	0.020	0.015
Exercise	s(Time of day):cluster2	2.36	2.31	2.71	2.64	0.055	0.063
Exercise	s(Time of day):cluster3	3.07	3.08	3.69	3.64	0.009	0.015
Exercise	s(Time of day):cluster4	1.00	1.00	1.36	1.36	0.243	0.244

Table S15. Comparison of smooth terms between primary GAMMs and AR(1) sensitivity models for behavioral EMA items

Effective Degrees of Freedom (EDFs), test statistics (F for time outside, socialized, and exercise and χ^2 for caffeine), and p-values are shown.

**Item**	**Term**	**EDF main model**	**EDF AR1 model**	**Test statistic main model**	**Test statistic AR1 model**	***p* main model**	***p* AR1 model**
Time outside	s(id)	236.46	238.00	1.43	1.45	0.000	0.000
Time outside	s(Time of day)	4.09	4.09	29.91	30.02	0.000	0.000
Time outside	s(Time of day):cluster1	1.00	1.00	2.13	2.12	0.145	0.146
Time outside	s(Time of day):cluster2	1.00	1.00	1.75	1.74	0.186	0.187
Time outside	s(Time of day):cluster3	1.00	1.00	1.88	1.88	0.170	0.171
Time outside	s(Time of day):cluster4	1.00	1.00	1.80	1.79	0.180	0.181
Socialized	s(id)	312.96	307.53	3.58	3.32	0.000	0.000
Socialized	s(Time of day)	4.32	4.30	3.93	3.89	0.003	0.003
Socialized	s(Time of day):cluster1	1.82	1.82	2.14	2.08	0.116	0.123
Socialized	s(Time of day):cluster2	1.00	1.00	2.55	2.48	0.110	0.116
Socialized	s(Time of day):cluster3	1.00	1.00	2.34	2.25	0.127	0.133
Socialized	s(Time of day):cluster4	1.00	1.00	3.20	3.12	0.074	0.078
Exercised	s(id)	285.97	290.80	2.50	2.64	0.000	0.000
Exercised	s(Time of day)	3.60	3.59	30.49	30.97	0.000	0.000
Exercised	s(Time of day):cluster1	1.00	1.00	0.31	0.31	0.575	0.580
Exercised	s(Time of day):cluster2	1.00	1.00	0.06	0.06	0.811	0.814
Exercised	s(Time of day):cluster3	1.00	1.00	0.13	0.13	0.717	0.719
Exercised	s(Time of day):cluster4	1.73	1.74	0.70	0.71	0.498	0.488

Table S16. Multivariate normality test

Mardia's skewness and kurtosis statistics and p-values for each cluster; Multivariate Normality Tests (MVN).

Cluster	Test	Statistic	p-value	MVN
Cluster 1: High appetitive motivation	Mardia Skewness	103.766	0.071	✓ Normal
Cluster 1: High appetitive motivation	Mardia Kurtosis	-1.534	0.125	✓ Normal
Cluster 2: Balanced	Mardia Skewness	110.421	0.028	✗ Not normal
Cluster 2: Balanced	Mardia Kurtosis	-0.501	0.616	✓ Normal
Cluster 3: Depressive with preserved goal-directed behavior	Mardia Skewness	81.269	0.564	✓ Normal
Cluster 3: Depressive with preserved goal-directed behavior	Mardia Kurtosis	-0.349	0.727	✓ Normal
Cluster 4: Depressive with low goal-directed behavior	Mardia Skewness	114.395	0.015	✗ Not normal
Cluster 4: Depressive with low goal-directed behavior	Mardia Kurtosis	-0.126	0.900	✓ Normal
